# Inhibition of redox/Fyn/c-Cbl pathway function by Cdc42 controls tumour initiation capacity and tamoxifen sensitivity in basal-like breast cancer cells

**DOI:** 10.1002/emmm.201202140

**Published:** 2013-04-22

**Authors:** Hsing-Yu Chen, Yin M Yang, Brett M Stevens, Mark Noble

**Affiliations:** 1 Department of Biomedical Genetics, University of Rochester Medical CenterRochester, NY, USA; 2 Department of Pathology and Laboratory Medicine, University of Rochester Medical CenterRochester, NY, USA; 3 Current address: StemgentSan Diego, CA, USA

**Keywords:** basal-like breast cancer, cancer stem cell, c-Cbl, redox/Fyn/c-Cbl pathway, tamoxifen

## Abstract

We found that basal-like breast cancer (BLBC) cells use Cdc42 to inhibit function of the redox/Fyn/c-Cbl (RFC) pathway, which normally functions to convert small increases in oxidative status into enhanced degradation of c-Cbl target proteins. Restoration of RFC pathway function by genetic or pharmacological Cdc42 inhibition enabled harnessing of pro-oxidant effects of low µM tamoxifen (TMX) concentrations – concentrations utilized in trials on multiple tumour types – to suppress division and induce death of BLBC cells *in vitro* and to confer TMX sensitivity *in vivo* through oestrogen receptor-α-independent mechanisms. Cdc42 knockdown also inhibited generation of mammospheres *in vitro* and tumours *in vivo*, demonstrating the additional importance of this pathway in tumour initiating cell (TIC) function. These findings provide a new regulatory pathway that is subverted in cancer cells, a novel means of attacking TIC and non-TIC aspects of BLBCs, a lead molecule (ML141) that confers sensitivity to low µM TMX *in vitro* and *in vivo* and also appear to be novel in enhancing sensitivity to a non-canonical mode of action of an established therapeutic agent.

## INTRODUCTION

Two of the most critical goals in cancer research are to identify means of increasing the utility of existing therapeutic strategies and of discovering new vulnerabilities of cancer cells. Such goals are most effectively achieved by the discovery of molecular mechanisms that play a role in both resistance to existing therapies and in other critical properties of cancer cells.

Tamoxifen (TMX) offers an exceptionally attractive target for such efforts, both because of the extensive experience with this agent and because of its two distinct modes of action. At mid-nanomolar (nM) exposure levels, TMX inhibits the division of oestrogen-dependent luminal breast cancer cells. Induction of cell death, however, requires exposure to micromolar (µM) concentrations of TMX. Such µM TMX concentrations are clinically relevant even with standard exposures to TMX [for which TMX concentrations in the tumour are ∼2 µM (Decensi et al, 1999; Johnston et al, 1993; Kisanga et al, 2004; Shin & Arteaga, 2006; Zhang et al, 1999; Zheng et al, 2007)], and are particularly important in attempts to utilize several-fold higher concentrations of TMX for treating a wide range of cancers, including gliomas (Hercbergs et al, 2003), lung cancer (Perez et al, 2003), metastatic melanoma (O'Day et al, 2001), desmoid tumours (Hansmann et al, 2004), non-Hodgkin's lymphoma (Ezzat et al, 2000), hormone refractory prostate cancer (Hamilton et al, 2003), hepatocellular carcinoma (Lu et al, 2004), cervical cancer (Ferrandina et al, 2001), metastatic renal cell carcinoma (Samuels et al, 1997) and metastatic uterine leiomyosarcoma (Yeh et al, 2003).

While relatively little is known about how cancer cells evade the pro-apoptotic effects of µM TMX, there appears to be enough clues to offer a speculative hypothesis as follows: It was recently reported that overexpression of the c-Cbl ubiquitin ligase enhanced the ability of µM TMX to induce apoptosis in oestrogen receptor-α (ERα) positive (and TMX-sensitive) MCF-7 luminal epithelial cancer cells, while expression of the dominant-negative (70Z) mutant of c-Cbl (DN(70Z)-c-Cbl) inhibited such effects (Yan et al, 2011). Other studies on ERα-negative MDA-MB 231 basal-like breast cancer (BLBC) cells have shown that Cdc42 can inhibit the ability of c-Cbl to promote degradation of one of its target proteins, the receptor for epidermal growth factor [EGFR, which is overexpressed in many BLBCs (Hirsch et al, 2006)]. What then might link these observations to the activities of TMX? One possible linkage is related to observations that µM TMX exposure makes cells more oxidized (Ferlini et al, 1999; Gundimeda et al, 1996; Mandlekar & Kong, 2001). When considered in light of our discovery that increased oxidative status (induced by environmental toxicants) leads to sequential activation of Fyn kinase and of c-Cbl, followed by enhanced degradation of c-Cbl targets (Li et al, 2007), the pro-oxidant effects of TMX offer a theoretical means of activating c-Cbl (Supporting Information Fig S1).

Hence, we investigated the novel hypothesis that cancer cells escape the pro-apoptotic effects of µM TMX by inhibiting c-Cbl activation via the redox/Fyn/c-Cbl (RFC) pathway, and that Cdc42 inhibition will enhance the utility of TMX as a potential treatment for ERα-negative cancer cells. We tested this hypothesis in basal-like breast cancer (BLBC) cells, because of previous studies on Cdc42 and c-Cbl interactions in such cells (Hirsch et al, 2006) and also because of the importance of developing improved treatments for these aggressive cancers that are resistant to the front-line breast cancer treatments of Herceptin® (Trastuzumab), TMX and aromatase inhibitors. Our findings provide a novel mechanism of TMX-mediated killing and TMX-resistance. Moreover, we provide a lead therapeutic candidate that pharmacologically enhances TMX sensitivity of BLBC cells *in vitro* and *in vivo*. In addition, these studies revealed that Cdc42-mediated inhibition of c-Cbl plays an unexpectedly critical role in enabling tumour generation by BLBC cells.

## RESULTS

### TMX fails to induce c-Cbl activation and reductions in EGFR levels in BLBC cells

**Figure 1:**
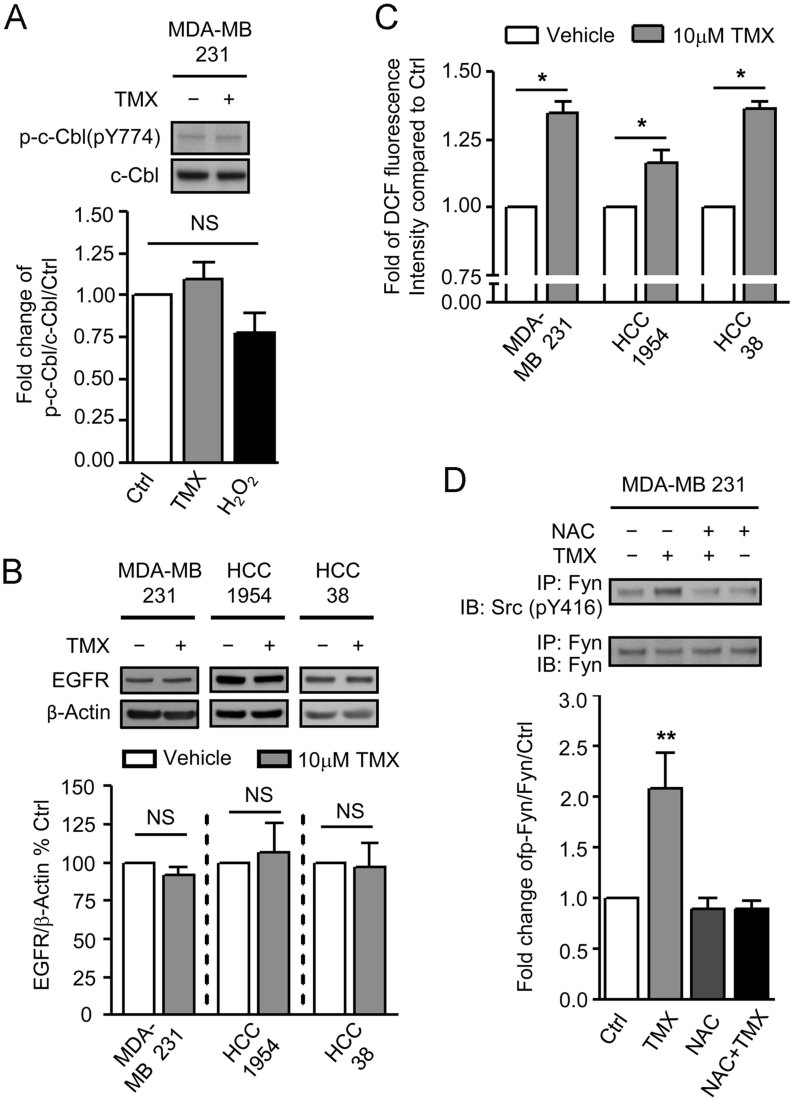
The RFC pathway is dysregulated in BLBC cells. A. Levels of phosphorylated c-Cbl at Tyrosine residue 774 and total c-Cbl were examined by immunoblotting of cells exposed to DMSO, 10 µM TMX or 200 µM H_2_O_2_ for 1 h. B. BLBC cells were exposed to DMSO or 10 µM TMX for 12 h. Levels of EGFR was examined by immunoblotting of these cells and quantified. C. BLBC cells were exposed to DMSO or 10 µM TMX for 1 h and subsequently levels of cytoplasmic ROS were determined by CM-H2DCFDA analysis. D. MDA-MB 231 cells were exposed to 10 µM TMX for 1 h w/ or w/o 12 h pretreatment with 2.5 mM NAC. Levels of Tyr-phosphorylated Fyn and total Fyn were examined by immunoblotting of immunoprecipiated Fyn against pSrc-416 and Fyn, respectively. Data represents Mean ± SEM (*n* ≥ 3). Student's *t*-test was performed to compare to control. *p < 0.05, **p < 0.01.

The key first prediction of our work was that even if low µM TMX can oxidize BLBC cells it is not able to cause activation of c-Cbl or reductions in levels of c-Cbl targets (with the EGFR examined as a relevant example of such targets). This hypothesis was found to be correct, such that exposure of the MDA-MB 231 BLBC cell line to 10 µM TMX or to H_2_O_2_ did not cause increases in c-Cbl phosphorylation or decreases in levels of EGFR. Similarly, exposure to 10 µM TMX did not cause any decreases in levels of EGFR in HCC1954 and HCC38 cells (Fig 1A and B).

The failure to activate c-Cbl and cause decreases in EGFR levels was not due to a failure to oxidize cells or to activate Fyn kinase. Exposure to 10 µM TMX caused 15–35% increases in oxidative status (as determined by labelling with DCF) in all three cell lines (Fig 1C), along with a >twofold increase in the proportion of oxidized glutathione in the total intracellular glutathione pool, from 10 to 23% (Supporting Information Fig S2). As predicted from our previous studies (Li et al, 2007), this increase in oxidative status was sufficient to cause a significant increase in the activation of Fyn kinase, an increase that was prevented by pre-treatment of cells with *N*-acetylcysteine (NAC, an anti-oxidant that also functions as a glutathione pro-drug; Fig 1D).

### TMX-induced activation of the RFC pathway in BLBC cells is prevented due to expression of Cdc42

**Figure 2:**
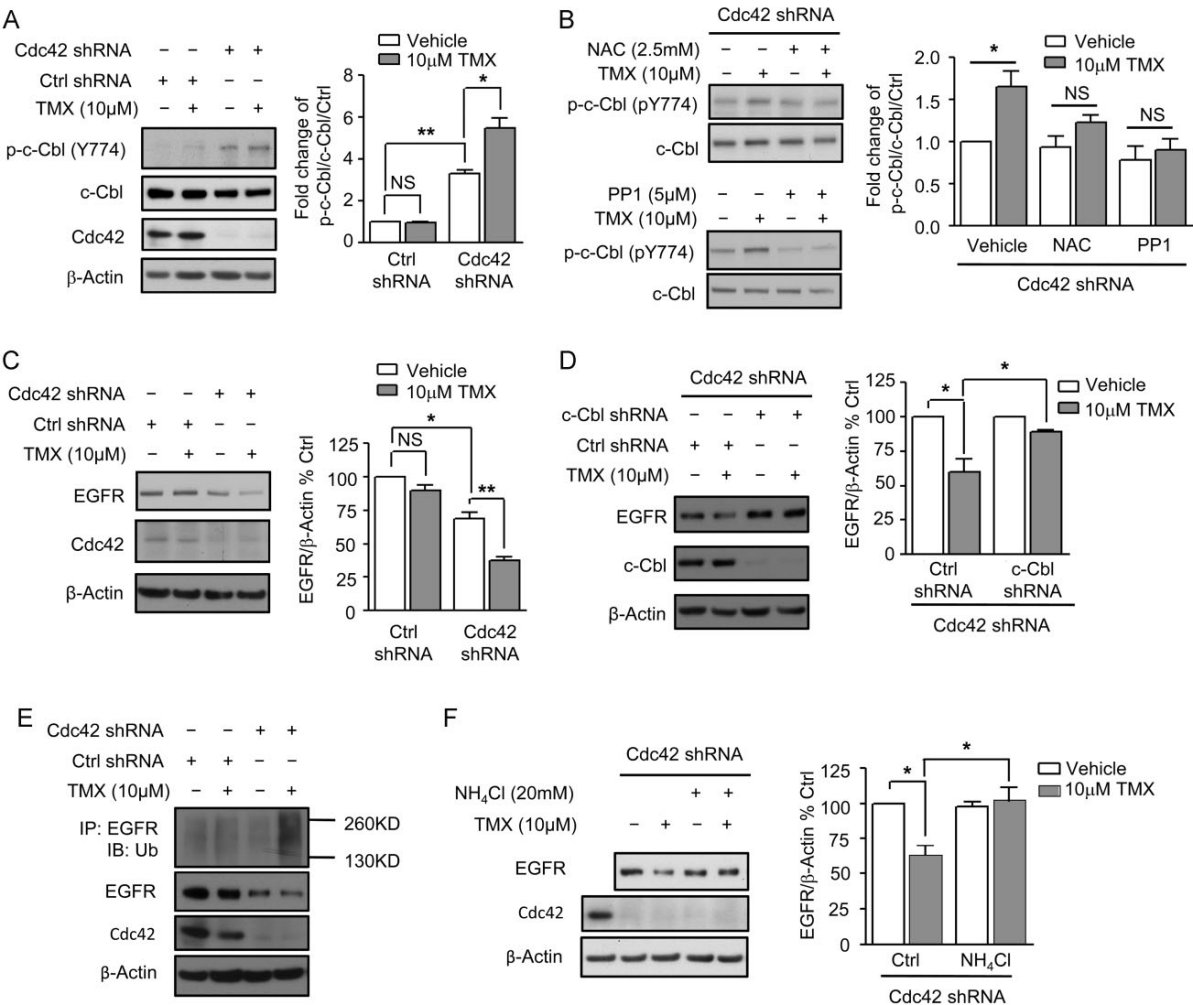
Inhibition of Cdc42 restores c-Cbl function in BLBC cells. A. MDA-MB 231 cells expressing scrambled or Cdc42 shRNAs were exposed to DMSO or to 10 µM TMX for 1 h and B. Cdc42 knockdown MDA-MB 231 cells pretreated with 2.5 mM NAC or 5 µM PP1 for 12 h were exposed to 10 µM TMX for 1 h. Levels of Tyr774-phosphorylated c-Cbl, total c-Cbl and Cdc42 were measured by immunoblotting of cells from (A,B). C–F. MDA-MB 231 cells expressing scrambled or Cdc42 shRNAs were exposed to DMSO or 10 µM TMX for 12 h and Cdc42 knockdown MDA-MB 231 cells further (D) expressing c-Cbl shRNAs or (F) co-treated with 20 mM NH_4_Cl were exposed to DMSO or 10 µM TMX for 12 h. Cells from (C, D and F) were harvested for measurement of EGFR levels normalized by expression of β-actin. (E) MDA-MB 231 cells expressing scrambled or Cdc42 shRNAs were exposed for 12 h to 10 µM TMX, after which ubiquitination of EGFR and total levels of EGFR of cells were measured. Data represents mean ± SEM (*n* ≥ 3). Student's *t*-test was performed. *p < 0.05, **p < 0.01.

If Cdc42 is responsible for blocking TMX-induced c-Cbl activation, then knockdown of Cdc42 should restore this activation and cause decreases in levels of the EGFR. When Cdc42 levels in MDA-MB 231 cells were reduced by expression of Cdc42 shRNA constructs, endogenous c-Cbl phosphorylation was increased by three- to fourfold [an outcome that seems likely due to the activation of Fyn kinase by oxidized glutathione (Hehner et al, 2000) and the finding that oxidized glutathione represents ∼10% of the total glutathione pool in these cells (GSSG; Supporting Information Fig S2), as compared with generally representing 2% of total glutathione in normal cells (Slivka et al, 1987)]. Moreover, exposure to 10 µM TMX now caused further increases in c-Cbl phosphorylation (Fig 2A). As found in our studies on environmental toxicants, this increase in c-Cbl phosphorylation was prevented by pre-treatment of cells with NAC or with PP1 [an inhibitor of Src family kinases, including Fyn kinase (Li et al, 2007)] (Fig 2B). These effects were specific to Cdc42, as genetic knockdown of levels of Cool-1 [which has been reported as a binding partner of Cdc42 (Schmidt et al, 2006)] in MDA-MB 231 cells did not alter Cdc42 activity and did not enable TMX exposure to induce c-Cbl phosphorylation (Supporting Information Fig S3).

As predicted by the activation of c-Cbl in Cdc42 knockdown cells, these cells also showed a decrease in EGFR levels (of ∼30%; *p* < 0.05), and TMX exposure caused a still greater decrease in levels of EGFR (of ∼60%; *p* < 0.01) in Cdc42 knockdown cells (Fig 2C).

The effects of Cdc42 knockdown were c-Cbl dependent, as demonstrated by expressing shRNA for c-Cbl in the Cdc42 knockdown cells. This secondary c-Cbl knockdown prevented TMX-induced decreases in levels of the EGFR, an effect not seen with scrambled shRNA (Fig 2D). The interpretation that c-Cbl was responsible for mediating TMX-induced EGFR degradation was further supported by the observations that exposure of MDA-MB 231 cells with reduced levels of Cdc42 to 10 µM TMX caused a marked increase in ubiquitination of EGFR as compared to scrambled controls (Fig 2E), and that the presence of 20 mM ammonium chloride (NH_4_Cl) – a specific lysosomal inhibitor – also suppressed TMX-induced reductions in EGFR levels in Cdc42 knockdown cells (Fig 2F), as predicted by our original studies identifying the redox/Fyn/c-Cbl pathway (Li et al, 2007).

### Pharmacological inhibition of Cdc42 with ML141 enhances sensitivity of BLBC cells to TMX

If the findings that Cdc42 inhibition enhances TMX sensitivity are going to be developed in a clinically applicable manner, then it is necessary to be able to inhibit Cdc42 pharmacologically. We pursued this goal by examining the utility of ML141, a selective Cdc42 inhibitor recently identified in a screen of inhibitors for small Rho family GTPases (Surviladze et al, 2010).

Pharmacological inhibition of Cdc42 with ML141 was similarly effective as genetic knockdown at enabling TMX-induced reductions in levels of EGFR. Co-exposure to 20 µM ML141 (a concentration that selectively inhibits Cdc42 function in BLBC cells; Supporting Information Fig S4) enabled TMX to cause a ∼45% reduction in EGFR levels (Supporting Information Fig S5).

**Figure 3:**
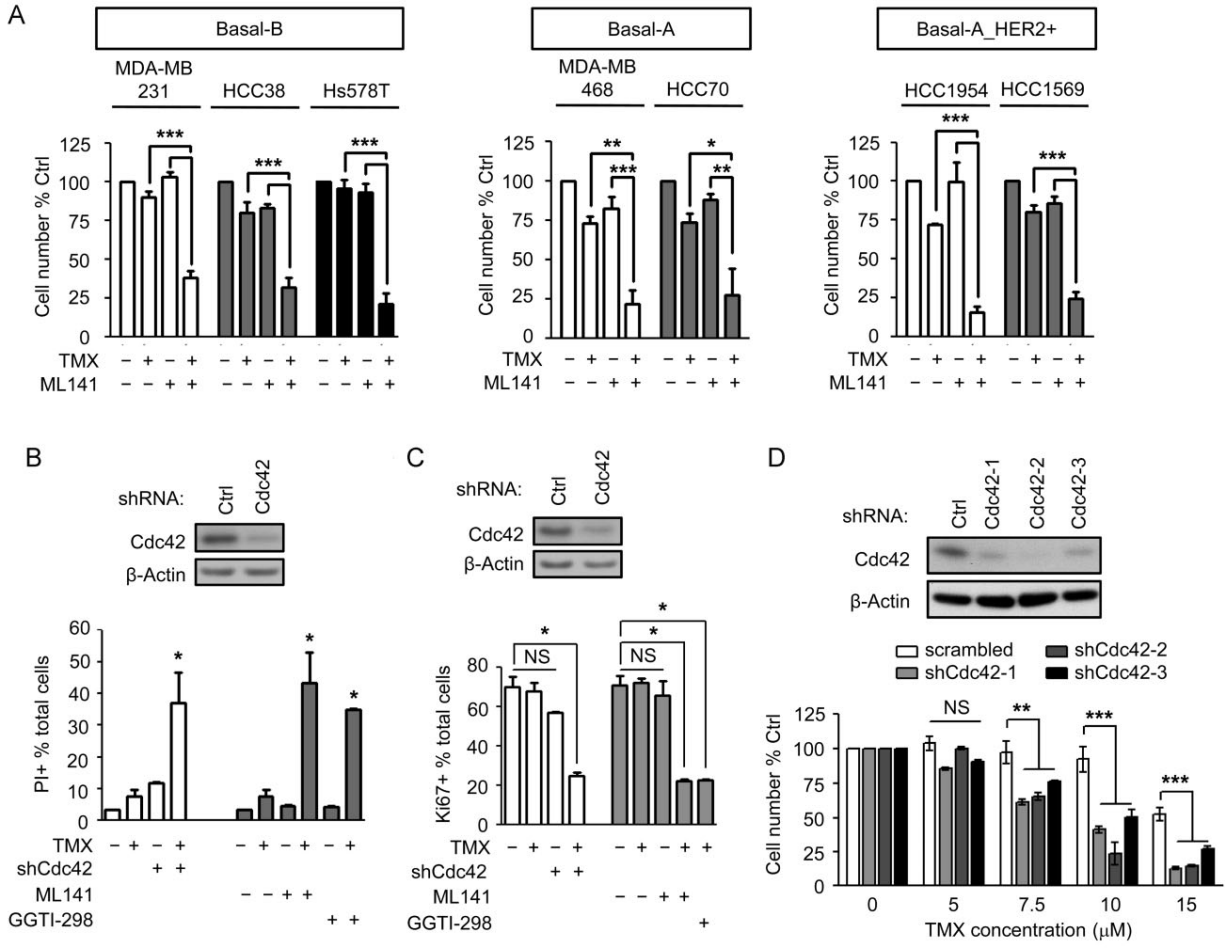
Inhibition of Cdc42 increases TMX sensitivity in BLBC cells. A. BLBC cell lines were exposed to 10 µM TMX, to 20 µM ML141 or both together for 48 h. B,C. Cell death and (C) cell division were determined by PI and Ki67, respectively, in MDA-MB 231 cells exposed to 10 µM TMX, to 20 µM ML141, to 5 µM GGTI-298 or to the combinations, or in Cdc42 knocked down cells treated with 10 µM TMX. Numbers represent the proportion of cells in the total cell pool. D. MDA-MB 231 cells treated with scrambled or Cdc42 shRNAs were exposed to increasing concentrations of TMX for 48 h. Viable cell number was measured by Calcein-AM + cell counting. Data represents mean ± SEM (*n* ≧ 3). Student's t-test was performed. *p < 0.05, **p < 0.01, ***p < 0.001.

ML141 exposure also enhanced the ability of TMX to suppress BLBC cell growth, through both induction of cell death and suppression of cell division. Exposure to 10 µM TMX alone caused 5–30% decreases in cell number in BLBC cell lines (including Basal-B: MDA-MB 231, HCC38 and Hs578T; Basal-A: MDA-MB 468 and HCC70 and Basal-A with HER2 amplification: HCC1954 and HCC1569), exposure to ML141 alone had no apparent effect on cell number, and exposure to ML141 + 10 µM TMX caused 70–90% decreases in cell number (Fig 3A). MDA-MB 231 cells exposed to 20 µM ML141, or Cdc42 knockdown cells, showed small increases in the proportion of dead cells in the cultures [as indicated by labelling of live cell cultures with propidium iodide (PI)]. In contrast, exposure to 10 µM TMX in Cdc42 knockdown cells or cells co-treated with ML141 caused an ∼10-fold increase in cell death as compared to control cell cultures, such that 35 or 43% of cells were now PI+, respectively (Fig 3B). Inhibition of Cdc42 also greatly enhanced TMX-induced suppression of cell division, as analysed by labelling cells with monoclonal Ki67 antibody. Cdc42 knockdown or treatment with ML141 enabled 10 µM TMX to cause a 45–50% reduction in the proportion of Ki67 + MDA-MB 231 cells, respectively (Fig 3C). These TMX concentrations are similar to those known to cause apoptosis, rather than inhibition of proliferation, in MCF-7 cells (Kallio et al, 2005). Similar results were obtained by treating cells with 5 µM GGTI-298 (which inhibits geranylgeranyltransferase I (GGTase I) function, thereby preventing prenylation of Rho-family GTPases (including Cdc42) and decreasing their function). In all these BLBC cell lines (except HCC70), exposure to GGTI-298 also enabled 10 µM TMX to cause ∼70 to 95% decreases in the cell number (Supporting Information Fig S6).

Similar decreases in cell number were seen when MDA-MB 231 cells expressing a scrambled shRNA construct were exposed to 10 µM TMX. In cells expressing any of three different shRNA constructs for Cdc42, in contrast, this concentration of TMX caused 50–75% reductions in cell number (*p* < 0.001 for all constructs). This effect was even greater in cells exposed to 15 µM TMX, which caused as much as 90% reductions in cell numbers (Fig 3D). In the Basal-A/Her2-amplified and slightly more sensitive HCC1954 cells, expression of Cdc42 shRNA also caused a greater response to TMX than seen in cells expressing scrambled shRNA (Supporting Information Fig S7). As observed in the case of TMX-induced reductions in EGFR levels, knockdown of Cool-1 also did not cause increased sensitivity to TMX as a cytotoxic agent in MDA-MB 231 cells (Supporting Information Fig S8).

**Figure 4:**
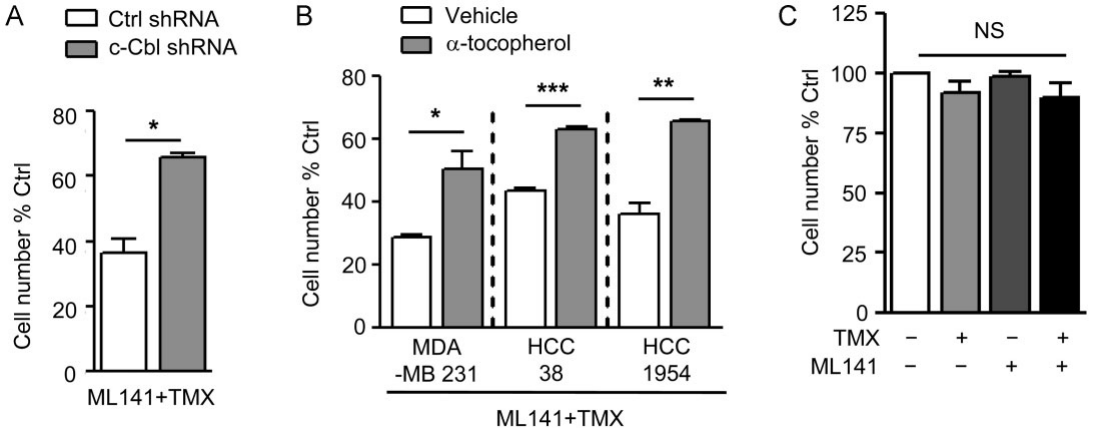
Increased TMX sensitivity caused by Cdc42 inhibition is oxidation and c-Cbl dependent. A. MDA-MB 231 cells expressing control or c-Cbl shRNAs were exposed to the combination of 10 µM TMX and 20 µM ML141 for 48 h. B. BLBC cell lines were exposed to 10 µM TMX ± 20 µM ML141 w/ or w/o concurrent treatment with 12.5 µM α-tocopherol for 48 h. C. MCF10A cells were exposed to 10 µM TMX, to 20 µM ML141 or the combination for 48 h. Viable cell number was measured by cell counts of Calcein-AM-positive staining of cells. Numbers represent the percentage change in cell number compared to un-dosed controls. Data represents mean ± SEM (*n* = 3). Student's *t*-test was performed. *p < 0.05, **p < 0.01, ***p < 0.001.

The outcomes seen in cells treated with ML141 + TMX were dependent on both Cdc42 inhibition and TMX exposure, and also were dependent on both c-Cbl and oxidation. Exposure to ML141 or TMX alone had no detectable effect on the proportion of Ki67+ cells. Moreover, MDA-MB 231 cells expressing c-Cbl shRNA were resistant to the combination of TMX and ML141, as compared to scrambled shRNA-treated cells receiving the same treatment (Fig 4A). In addition, co-exposure to 12.5 µM α-tocopherol (a soluble analogue of the anti-oxidant Vitamin E) prevented reductions in cell number caused by exposure to the combination of TMX + ML141, as examined in both Basal-B (MDA-MB 231 and HCC38) and Basal-A with HER2 amplification (HCC1954) cells (Fig 4B).

Despite the increased toxicity of TMX for BLBC cells in which Cdc42 function was inhibited, such inhibition did not increase TMX toxicity on non-tumourigenic mammary epithelial cells. When MCF10A cells were exposed to 10 µM TMX, 20 µM ML141 or the combination, no reduction in cell number was detected (Fig 4C). Thus, effects of Cdc42 inhibition on TMX-responsiveness appeared to be specific to BLBC cells.

### Genetic or pharmacological inhibition of Cdc42 enables *in vivo* treatment of MDA-MB 231 cells with TMX

In light of the ability of Cdc42 inhibition to confer TMX sensitivity of BLBC cells *in vitro*, we next examined the effects of such inhibition on responsiveness of BLBC cells to TMX *in vivo*. In these experiments, animals were transplanted with 1 × 10^6^ cells, yielding tumours in 100% of mice. After 20 days of tumour growth, TMX releasing pellets (7.5 mg/60 days) were implanted subcutaneously, thus mimicking clinical scenarios in which a tumour mass has already grown prior to initiation of treatment. As a wide range of values have been reported for the concentrations of serum TMX following implantation of such pellets, as well as for TMX concentrations in the tumour, our central concern was to use a dosage that would not exceed tumour levels occurring during utilization of high-dose TMX for treatment of tumours in humans.

**Figure 5:**
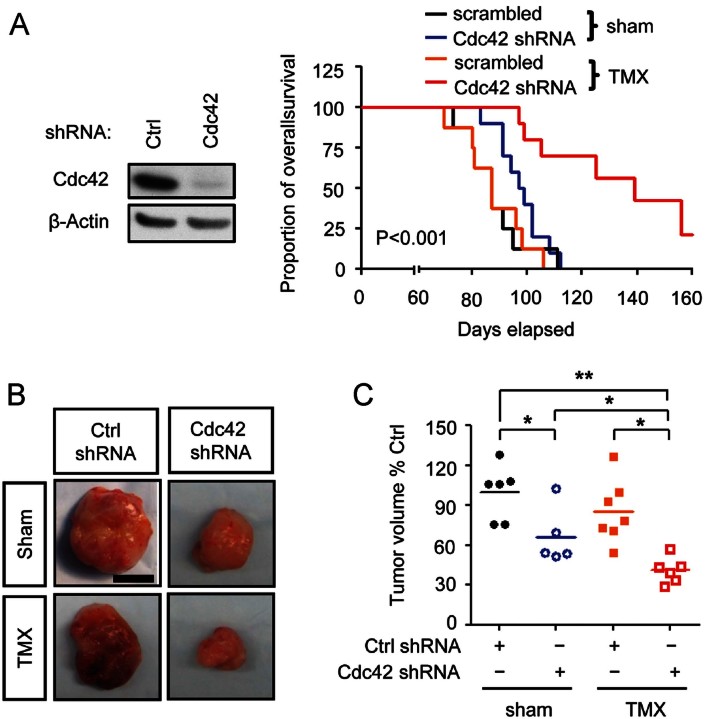
Reduction of Cdc42 expression increases TMX sensitivity of BLBC cells *in vivo*. To understand if reducing Cdc42 levels increases BLBC cell sensitivity to TMX *in vivo*, for each xenograft, 1,000,000 cells were transplanted into mice, and at Day 20 after transplantation, a TMX releasing pellet (7.5 mg/60 days) was subcutaneously implanted. A. The survival of mice with breast tumours was recorded and analysed by Mantel–Cox test. The p value refers to the comparison among all groups. B,C. At Day 60, breast tumours were harvested and photographed and tumour volume was determined (C). Scale bar represents 0.5 cm. One-way ANOVA followed by Bonferroni pair-wise comparison was performed. *p < 0.05, **p < 0.01.

When tumours were generated from Cdc42 knockdown cells, these tumours showed increased responsiveness to TMX *in vivo*, as compared with tumours derived from cells expressing scrambled shRNAs (Fig 5). The median survival time of mice transplanted with MDA-MB 231 cells expressing scrambled shRNAs was 87 days, and was essentially unchanged if such animals were treated with TMX. Mice transplanted with Cdc42 knockdown cells showed a small increase in median survival to 98 days. Animals transplanted with Cdc42 knockdown cells and treated with TMX showed a greater extension of survival, with no deaths occurring until most animals (7/8) transplanted with cells expressing scrambled constructs had already died at 98 days. At the time when all the animals in all other groups had died (including mice transplanted with Cdc42 knockdown cells, the last of which died at 112 days), almost half of the animals harbouring Cdc42 knockdown cells and treated with TMX remained alive until 140 days (Fig 5A). Such effects appeared to be c-Cbl dependent, as systemic TMX administration failed to extend the survival time of animals implanted with cells expressing both c-Cbl and Cdc42 shRNAs. The median survival time of mice transplanted with cells expressing c-Cbl and Cdc42 shRNAs was 96 days, and was unchanged (98 days) when such animals were treated with TMX (Supporting Information Fig S9).

Cdc42 knockdown and TMX treatment also showed modest effects on tumour volume as measured after 60 days of tumour growth. Cdc42 knockdown cells formed significantly smaller tumours than cells expressing scrambled shRNA (Fig 5B and C). TMX treatment was associated with a slight but not significant decrease in tumour volume in cells expressing scrambled shRNA. The smallest tumours were found in mice transplanted with Cdc42 knockdown cells and treated with TMX, with 5/6 tumours being smaller than any of the tumours found in any other experimental group (*p* < 0.05).

**Figure 6:**
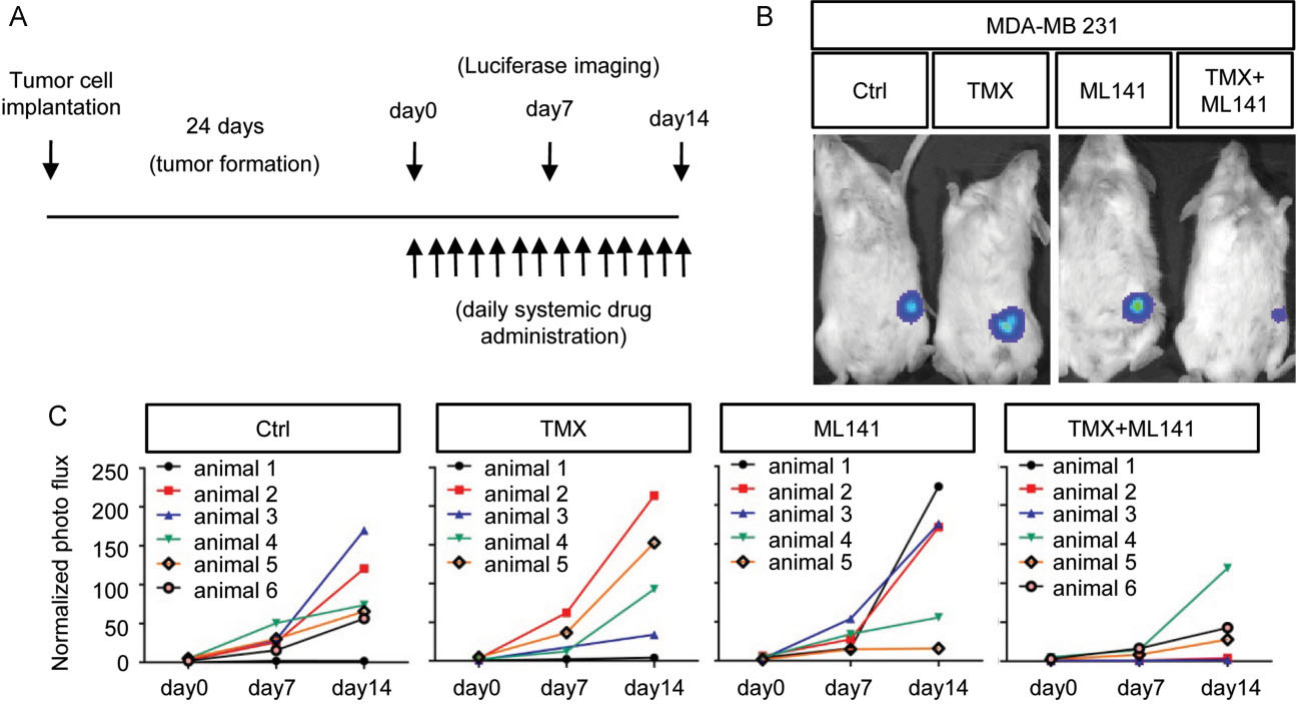
Pharmacological inhibition of Cdc42 enhances TMX sensitivity of BLBC cells *in vivo*. A. The regimen for examination of whether pharmacologically inhibiting Cdc42 activity increases BLBC cell sensitivity to TMX *in vivo* was that each mouse was transplanted with 1,000,000 cells and tumours were allowed to grow for 24 days. B,C. From Day 24 after transplantation, daily systemic drug administration started and continued for 14 days, and tumour size was monitored by whole mouse imaging every 7 days (B) and is displayed quantitatively in (C).

We were also able to apply ML141 *in vivo*, even though the original identification of this compound was not associated with selection for favourable properties *in vivo*, and its solubility characteristics only allowed analysis over a 2-week period *in vivo*, slightly longer than used in previous analyses of notch inhibitors (Rizzo et al, 2008). In these experiments, animals were transplanted with 1 × 10^6^ unmanipulated MDA-MB 231 cells and tumours were allowed to establish for 24 days before initiating treatment (Fig 6A). After confirming tumour establishment by luciferase imaging, animals were treated with 1 mg/day ML141, 125 µg/day TMX or both agents together, with ML141 delivered intraperitoneally in a corn oil vehicle.

Our experiments demonstrated that, even without optimization for *in vivo* utilization, pharmacological inhibition of Cdc42 with ML141 enabled TMX to suppress growth of MDA-MB 231 derived tumours. Remarkably, considering tumours were generated from a TMX-resistant BLBC cell line, exposure to TMX + ML141 was associated with a marked suppression of tumour growth during the 2 weeks of treatment (Fig 6B and C and Supporting Information Fig S10). In mice treated with vehicle only, 5/6 tumours increased markedly in size over these 2 weeks and one mouse showed no tumour growth. Neither TMX nor ML141 altered this outcome when applied individually. When both agents were combined, however, now only 1 out of 6 animals exhibited a marked increase in tumour size, 2/6 mice showed only modest tumour growth and 3/6 mice showed no tumour growth at all (Table 1).

**Table 1:** TMX in combination with ML141 *in vivo* suppresses BLBC cell growth

	Normalized photon flux				
	0–50	50–100	100–150	150–200	200–250
Day 0					
Ctrl	6/6	–	–	–	–
TMX	5/5	–	–	–	–
ML141	5/5	–	–	–	–
TMX + ML141	6/6	–	–	–	–
Day 7					
Ctrl	5/6	1/6	–	–	–
TMX	5/6	1/5	–	–	–
ML141	5/6	1/5	–	–	–
TMX + ML141	6/6	–	–	–	–
Day 14					
Ctrl	1/6	3/6	1/6	1/6	–
TMX	2/5	1/5	–	1/5	1/5
ML141	1/5	1/5	–	2/5	1/5
TMX + ML141	5/6	–	1/6	–	–

Animals were transplanted with 1,000,000 cells and treated with TMX, ML141 or the combination for 14 days. Tumour progression was monitored by whole mouse imaging every 7 days till completion of drug treatment.

### Reduction of Cdc42 expression reduces mammosphere formation *in vitro* and tumour initiation *in vivo*

In the course of defining the numbers of cells to transplant for our *in vivo* experiments, we observed that Cdc42 knockdown was associated with a reduction in the number of tumours generated in mice transplanted with fewer cells. These observations caused us to further look at the effects of Cdc42 inhibition on properties associated with TICs. As there exists continued debate regarding the utility of specific antigens in defining cells with the ability to initiate tumours, we focused attention on the ability to grow as adhesion-independent spheroids (also referred to as mammospheres) and to initiate tumours *in vivo*. In both cases, Cdc42 knockdown markedly inhibited the function examined.

**Figure 7:**
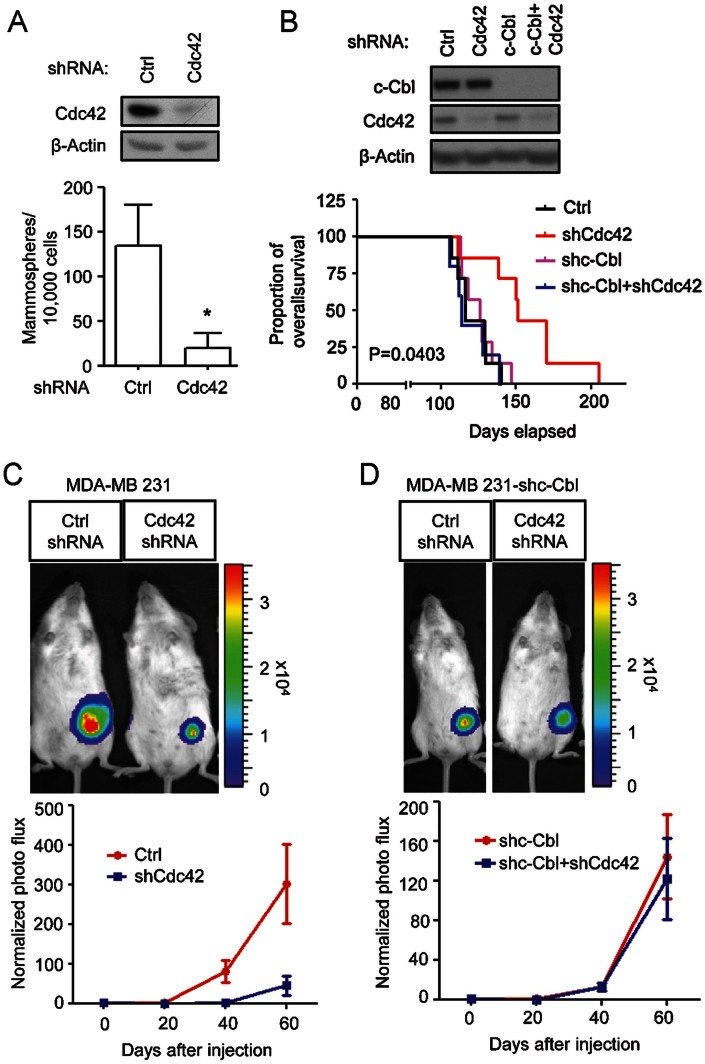
Knockdown of Cdc42 reduces *in vitro* mammosphere formation and inhibits tumour growth *in vivo* A. Ten thousand MDA-MB 231 cells expressing scrambled or Cdc42 shRNAs were plated on non-adherent plastic and supplemented with 20 ng/ml EGF and bFGF. The number of mammospheres with a sphere size >60 µm was counted at 7 days after seeding. B. Overall survival of mice with breast tumours was recorded and analysed by Mantel–Cox test. The p value refers to the comparison among all groups. C,D. For each xenograft *in vivo*, 100,000 cells were transplanted into the mammary fat pad of a female NOD/SCID mouse. (C) Scrambled or Cdc42 knockdown MDA-MB 231 cells were transplanted. (D) MDA-MB 231 cells expressing c-Cbl and/or Cdc42 shRNAs were transplanted. Tumour sizes were tracked and recorded by live imaging over time till Day 60 after transplantation.

As shown in Fig 7A, Cdc42 inhibition greatly decreased the ability of MDA-MB 231 cells to generate adhesion-independent spheroids. MDA-MB 231 cells expressing scrambled shRNAs generated ∼130 spheres per well when plated at 10,000 cells/well while cells expressing shRNAs for Cdc42 only generated ∼20 spheres per well.

The most important indicator of TIC function is the ability to generate tumours *in vivo*, and examination of this parameter revealed suppression of tumour initiation by Cdc42 inhibition in three different BLBC cell lines. In these experiments, varying numbers of MDA-MB 231 cells stably expressing Cdc42 shRNAs or scrambled shRNAs (which also expressed firefly luciferase) were injected orthotopically into female mouse mammary fat pads. Readily detectable tumours developed in ∼80% of NOD/SCID mice within 3 months after transplantation with as few as 1000 MDA-MB 231 cells treated with scrambled vectors, and mice transplanted with 10,000 cells all generated tumours. In striking contrast, when Cdc42 levels were decreased by shRNA expression, tumour take was reduced, with 1000 and 10,000 MDA-MB 231 cells establishing tumours in only one out of five and three out of eight NOD/SCID mice, respectively (Table 2; *p* < 0.05). Cdc42 knockdown was even more effective at preventing tumour generation by HCC1954 (Basal-A with HER2 amplification) and HCC70 (Basal-A) cells, and no cells expressing shRNA for Cdc42 generated tumours even when 10,000 cells were transplanted. Comparable transplants with cells expressing scrambled shRNA caused tumour generation of 60% of recipient mice.

**Table 2:** Cdc42 knockdown decreased the tumourigenic capacity of BLBC cells in a c-Cbl dependent manner

	Number of cells injected			
	1 × 10^6^	1 × 10^5^	1 × 10^4^	1 × 10^3^
MDA-MB 231				
Ctrl shRNA	6/6	5/5	7/7	4/5
Cdc42 shRNA	8/8	5/5	3/8*	1/5*
c-CblshRNA				
Ctrl shRNA	–	5/5	5/6	5/5
Cdc42 shRNA	–	5/5	5/8	3/5
HCC1954				
Ctrl shRNA	–	5/5	3/5	–
Cdc42 shRNA	–	4/5	0/5*	–
HCC70				
Ctrl shRNA	–	–	2/3	–
Cdc42 shRNA	–	–	0/2	–

Animals were transplanted with the number of cells indicated, and tumour progression was monitored by whole mouse imaging every 20 days till three months post transplantation.

* *p* < 0.05 in comparison of tumour development between animals xenografted with cells expressing scrambled or Cdc42 shRNAs.

To determine whether pharmacological inhibition of Cdc42 with ML141 affects TIC properties of BLBCs, 10,000 MDA-MB 231 cells pre-exposed for 40 h either to DMSO or 20 µM ML141 were implanted into animals. At 20 days after transplantation, a time when four out of five mice generated tumours from DMSO-treated cells, none of the animals implanted with ML141 pre-treated cells formed tumours (Supporting Information Fig S11A). When examined 40 days after implantation, two out of five mice harbouring ML141 pre-treated cells started to form tumours (a comparable frequency as with Cdc42 knockdown cells; Table 2). Moreover, the tumour sizes resulting from ML141 pre-treated cells were markedly smaller than those of the control group (Supporting Information Fig S11B).

Even when we transplanted 100,000 MDA-MB 231 cells with a prior Cdc42 knockdown we found that tumours grew more slowly and that animals showed longer survival. In animals transplanted with Cdc42 knockdown MDA-MB 231 cells, the average size of the tumour mass at 60 days after engraftment was only ∼20% the size of tumours generated from cells transduced with scrambled shRNA constructs (Fig 7C). Similar results were obtained with HCC1954 cells (Supporting Information Fig S12). The mean survival time of animals transplanted with Cdc42 knockdown MDA-MB 231 cells also was increased from 116 days in mice implanted with 100,000 scrambled shRNA-containing cells out to 151 days (Fig 7B). Moreover, at the time when all animals transplanted with scrambled shRNA expressing cells were dead, most of the mice transplanted with Cdc42 knockdown cells were still alive.

Confirmation that the *in vivo* effects of Cdc42 knockdown were c-Cbl-dependent was provided by transducing Cdc42 knockdown cells with secondary c-Cbl shRNAs before transplantation. Reduction of c-Cbl expression abolished the effects of Cdc42 knockdown on tumour initiation. When mice were transplanted with 10,000 Cdc42 knockdown cells that also expressed a secondary c-Cbl knockdown, the frequency of tumours increased from 38 to 63%, while in mice transplanted with 1000 such cells the tumour frequency increased from 20 to 60% (Table 2). Moreover, the decreased tumour size and prolonged survival seen in mice transplanted with 100,000 Cdc42 knockdown cells was dependent on restoration of c-Cbl function. When mice were transplanted with cells that co-expressed shRNAs for Cdc42 and c-Cbl the rate of tumour growth and the time of survival were indistinguishable from mice transplanted with cells expressing scrambled shRNA for Cdc42 (Fig 7B and D). Moreover, Cdc42-mediated inhibition of c-Cbl function was apparently so effective that expression of shRNA for c-Cbl in MDA-MB 231 cells expressing scrambled shRNA constructs did not cause any further increases in rate of tumour growth or decreases in time to death.

## DISCUSSION

**Figure 8:**
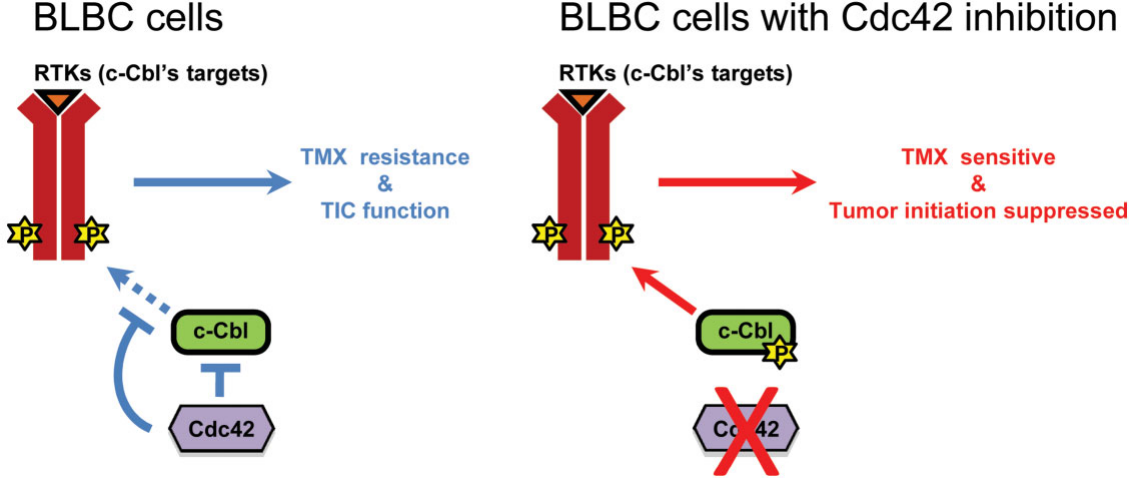
Schematic summary of the role of Cdc42 inhibition in the biology of BLBC cells. TMX resistance and tumour initiation in BLBC cells is due, at least in part, to inhibition of c-Cbl activation. Restoration of c-Cbl function, by perturbation of Cdc42, enables increased phosphorylation of c-Cbl, enhanced degradation of c-Cbl targeted RTKs (such as the EGFR), increased sensitivity to TMX and loss of TIC function.

The exploitation of the ability of low µM TMX to induce cancer cell apoptosis in an ERα-independent manner has made this agent of potential interest in the treatment of more than a dozen different types of cancers, but there has been little understanding of either how cells evade such effects or how to enhance the efficacy of these approaches. Our studies on this problem have led us to several novel discoveries that extend far beyond the specific concern of enhancing the utility of TMX. We found that BLBCs inhibit activity of the RFC pathway via Cdc42 and that restoring activity of this pathway by genetic or pharmacological inhibition of Cdc42 enables the pro-oxidant activities of low µM concentrations of TMX to be harnessed so as to have multiple beneficial effects on BLBCs, one of the most dangerous categories of breast cancers. These studies provide a new mechanism underlying resistance of BLBC cells to the ERα-independent effects of TMX, mechanism-driven approaches for overcoming such resistance, and a pharmacological lead candidate that enables treatment of ERα-negative BLBC cells *in vivo* with TMX. In addition, our studies provide novel approaches to inhibiting TIC function in these cells and novel insights into how cancer cells escape the consequences of increased oxidative status. Thus, these studies identify a single molecular pathway (summarized in Fig 8) that enables suppression of tumour initiation by BLBC cells and that also renders established BLBC tumours responsive to treatment with µM concentrations of TMX. This represents one of the only pathways thus far discovered that appears integral to both resistance to therapeutic strategies and integral to the biology of TICs.

Our present studies offer the first evidence of the ability of cancer cells to subvert function of c-Cbl's regulation via the RFC pathway. Critically, our data demonstrates that it is not the ability of TMX to render cells more oxidized that was critical to our outcomes, but the ability of such pro-oxidant effects to cause activation of c-Cbl via the RFC. This provides a new mechanism by which tumour cells can escape the effects of increases in oxidative status. These discoveries have potentially important implications in regards to increasing interests in targeting cancer cells through manipulation of redox state (see, *e.g.* Montero & Jassem, 2011; Vera-Ramirez et al, 2011). It has been observed repeatedly that cancer cells exhibit high levels of reactive oxidative species (ROS), despite often also expressing increased levels of proteins involved in decreasing oxidative stress. In contrast with managing production of oxidative species themselves, however, inhibition of the RFC pathway provides a mechanism that enables cancer cells to escape the functional consequences of being more oxidized.

One important aspect of restoration of function of the RFC pathway is that it appears to affect both putative TICs and non-TICs. Many agents that are used to treat tumours have little or no effect on TICs, and the resistance of these cells to mainstream cancer treatments is thought to be one of the major factors limiting the efficacy of old and new therapies (see, *e.g.* Al-Ejeh et al, 2011; O'Brien et al, 2011). Our studies appear to represent one of only three pathways identified thus far that strongly suppress tumour initiation activity through mechanisms relevant to BLBCs, with the other two being inhibition of Notch signalling and of the CXCR1 chemokine receptor. Genetic and pharmacological inhibition of Notch signalling decreased tumour generation by oestrogen-dependent MCF 7 cells and BLBC-derived MDA-MB 231 cells (Harrison et al, 2010; Rizzo et al, 2008) and pharmacological inhibition of gamma secretase (thus inhibiting Notch activation) suppressed tumour growth for 10 days when treatment was started at the time of cell transplantation. Pharmacological inhibition of CXCR1 with repertaxin also caused a marked decrease in mammosphere formation *in vitro* and targeted TICs *in vivo* (Ginestier et al, 2010).

Restoration of c-Cbl function also appears to be effective at targeting non-TICs, a particularly important concept as it is becoming increasingly clear that the distinction between TICs and non-TICs is a plastic one and that cells in the putative non-TIC compartment also can maintain and initiate tumours. Based on the observations that growth of 10,000 MDA-MB 231 cells *in vitro* yielded ∼130 mammospheres, and transplantation of 1000 such cells yielded tumours in most but not all mice, this suggests a TIC frequency in the range of ∼0.1 to 1%. Yet, in cells with Cdc42 knockdown or treated with ML141 and exposed to TMX (Fig 3), cell death *in vitro* increased from ∼3% of cells in untreated cultures to ∼40% of cells, while the proportion of Ki67+ cells fell by two-thirds (from ∼75% in untreated cultures to ∼25% in cultures receiving both treatments). Moreover, treatment with ML141 + TMX caused a suppression of further tumour growth *in vivo* (Fig 6 and Supporting Information Fig S10). Such outcomes argue for effects that extend beyond the small fraction of cells with TIC function in these populations. When taken together with the ability of restoration of c-Cbl function via Cdc42 knockdown to inhibit mammosphere formation and tumour initiation *in vivo*, it appears that such manipulations modulate the biology of both TICs and of the bulk population of BLBC tumour cells. This outcome thus compares favourably with the effects of repertaxin, which causes death also of CXCR1-negative cells by promoting secretion of Fas ligand (Ginestier et al, 2010).

Our findings also extend the understanding of potential contributions of Cdc42 to tumour biology in several ways. Cdc42 has mostly been studied as a regulator of the actin cytoskeleton and for its role in generating actin-rich protrusions (called invadopodia) and, in cancer biology, as facilitating cancer cell migration and metastasis (Fritz & Kaina, 2006; Hirsch et al, 2006). It also has been observed, in over-expression experiments, that a complex of Cdc42 and Cool-1 can bind to c-Cbl, leading to a failure of normal downregulation of the EGFR (Schmidt et al, 2006; Wu et al, 2003) and that reducing levels of Cdc42 in MDA-MB 231 cells can reduce cell proliferation and migration. Our results suggest, however, that Cdc42 also can inhibit TMX-induced c-Cbl activity independently of Cool-1, as knockdown of Cool-1 had no effect on phosphorylation of c-Cbl or on TMX sensitivity (Supporting Information Figs S3 and S8). The reasons for these differences are not clear, but the failure of Cool-1 knockdown to alter outcomes in our experiments was consistently observed. It is possible, however, that there are other aspects of Cdc42 inhibition of c-Cbl that may be relevant to our findings. High levels of Cdc42-interacting protein 4 (CIP4) in invasive breast cancer cells (Pichot et al, 2010), such as in BLBCs, might offer an alternative means by which Cdc42 inhibits c-Cbl function. It was previously reported that CIP4 interacts with c-Cbl via a Src Homology 3 domain (Dombrosky-Ferlan et al, 2003). We found that knockdown of CIP4 in MDA-MB 231 cells also caused a marked decrease in EGFR levels and that exposure of these CIP4 knockdown cells to TMX caused a further decrease in EGFR levels (Supporting Information Fig S13). Thus, investigation of CIP4 as a component of this circuitry might prove of interest in future investigations.

Our studies on ML141 appear to offer the first demonstration that pharmacological inhibition of Cdc42 is of potential use in cancer treatment, at least in the context of enhancing response to other agents. Pharmacological inhibition of the beta subunit of GGTase I with GGTI-298 has been shown previously to reduce tumour cell division by inhibiting geranylgeranylation of multiple Rho GTPase family members (Sun et al, 2003; van de Donk et al, 2003). ML141, in contrast, is the first direct and selective Cdc42 inhibitor (Surviladze et al, 2010) that appears to be useful as an adjunct therapy. It was also of interest in this regard that ML141 was not toxic for non-transformed MCF10A basal-like cells, nor did it render these cells sensitive to TMX, opening up the possibility that this compound exhibits at least some selectivity for cancer cells. Although the parental structure is not suitable for longer-term application than the 14-day time point used in our present studies, the demonstration of *in vivo* utility of the existing compound suggests that ML141 is an excellent lead candidate for further development.

Restoration of the ability of TMX to activate c-Cbl via the RFC pathway is an attractive target for cancer intervention for multiple reasons. The proteins that are ubiquitylated by c-Cbl include several receptors of particular interest in multiple cancers, including different types of breast cancer. EGFR expression is frequently elevated in malignant breast cancers, including BLBCs, and such increased expression is thought to contribute to chemoresistance, aggressive growth and expression of tumour initiating ability (Dawood, 2010; Foley et al, 2010; Saxena & Dwivedi, 2012). Inhibition of EGFR activity is of great interest as a treatment strategy for multiple cancers, but the reductions in EGFR levels that occur when c-Cbl function is restored – and are further enhanced by exposure to TMX – may achieve similar purposes. Restoring c-Cbl activity is likely to extend, however, beyond regulation of the EGFR. For example, mammosphere generation by ductal carcinoma *in situ* cells *in vitro* was suppressed by about 60% by Gefitinib (Farnie et al, 2007) [which inhibits EGFR but also inhibits multiple other kinases (Brehmer et al, 2005)] but there appears to be no evidence of suppression of tumuor generation or ability to render ERα-negative cells sensitive to TMX with this agent. Beyond the EGFR, Notch1 is regulated by c-Cbl activity (Jehn et al, 2002), thus providing the possibility of targeting this regulator of TIC activity (Harrison et al, 2010; Rizzo et al, 2008) via the RFC pathway. Moreover, the c-Met receptor for hepatocyte growth factor/scatter factor, which appears to be of significant importance in BLBCs (Gastaldi et al, 2010), is also a target of c-Cbl-mediated regulation (Li et al, 2007; Taher et al, 2002). Although HER2/Neu is not expressed in BLBCs, it is an important therapeutic target in other breast cancers (Arteaga et al, 2011; Hurvitz et al, 2013; Stern, 2012), and is itself a target of c-Cbl regulation (Levkowitz et al, 1996). Thus, restoration of RFC pathway function may provide beneficial effects on multiple pathways of increasing interest in controlling cancer cells. Moreover, given the role of receptors such as EGFR, c-Met and HER2 in activating signal transduction pathways, it is not surprising that activation of c-Cbl is also associated with reduced levels of Akt and NF-κB activity (Li et al, 2007), two additional targets of current cancer therapeutic strategies (see, *e.g.* De Luca et al, 2012; Morrow et al, 2011; Nogueira et al, 2011). Thus, while the potential role of c-Cbl as a tumour suppressor gene is of increasing interest (for review see, *e.g.* Dikic & Schmidt, 2007; Lipkowitz & Weissman, 2011), our studies provide new perspectives on ways in which inhibiting c-Cbl function can contribute to tumour cell function.

Our studies raise the possibility that the non-ERα-mediated effects of TMX may be able to be harnessed for therapeutic benefit by inhibiting Cdc42-mediated suppression of RFC pathway function. Our discoveries appear thus far to be unique in conferring sensitivity to a secondary mode of action of an established therapeutic agent, thus extending the potential use of TMX to a group of tumours that are otherwise non-responsive to this compound. In contrast with the well-studied utilization of TMX as an ERα inhibitor, the cells we transplanted did not express detectable ERα (Supporting Information Fig S14), and our *in vitro* analyses indicate these cells instead were responsive to the pro-oxidant effects of TMX. Thus, this is a qualitatively different outcome from the ability of Notch signalling inhibition to increase the sensitivity of ERα-expressing cells to TMX (Rizzo et al, 2008). The development of Cdc42 inhibitors suitable for use in the clinic could prove of considerable therapeutic value, as levels of Cdc42 in breast tumour lysates exceed by as much as 50-fold those seen in normal tissue from the same patients and elevated levels of Cdc42 also have been observed in lung cancer and in colorectal cancer (Fritz et al, 1999, 2002). Such increases in Cdc42 levels may serve as predictors of situations in which Cdc42 inhibition and exposure to TMX can be usefully applied for therapeutic purposes.

## MATERIALS AND METHODS

### Reagents and antibodies

Tamoxifen citrate, PI, α-tocopherol, NAC and corn oil were obtained from Sigma–Aldrich (St. Louis, MO, USA). Calcein-AM and CM-H2DCFDA were obtained from Invitrogen (Grand Island, NY, USA). GGTI-298 and ML141 were obtained from Tocris (Ellisville, MO, USA). PP1 was obtained from Calbiochem (San Diego, CA, USA). Anti-Fyn polyclonal, anti-Cdc42 monoclonal, anti-ubiquitin monoclonal and anti-β-Actin monoclonal antibodies were obtained from Santa Cruz Biotechnology (Santa Cruz, CA, USA). Anti-c-Cbl monoclonal, anti-CIP4 monoclonal and anti-Ki67 monoclonal antibodies were obtained from BD PharMingen (San Diego, CA, USA). Anti-Cool-1 polyclonal antibody was obtained from Millipore (Billerica, MA, USA). Anti-phosphorylated c-Cbl (Y774) polyclonal, anti-phosphorylated Src (Y416) polyclonal, anti-ERβ polyclonal and anti-EGFR polyclonal antibodies were obtained from Cell Signaling Technology (Beverly, MA, USA). Anti-ERα monoclonal antibody was obtained from Dako (Carpinteria, CA, USA).

### Constructs, viral packaging, cell infection and selection

All DNA-based short hairpin RNAs (shRNAs) in the pLKO.1-puro lentiviral expression vector were obtained from Open Biosystems (Huntsville, AL, USA). pLKO.1-c-Cbl, pLKO.1-Cdc42, pLKO.1-Cool-1, pLKO.1-CIP4 or the corresponding scrambled shRNA plasmids were co-transfected with VSV and PAX2 lentiviral packaging protein expression vectors into HEK 293TN cells by Fugene6 (Roche) transfection solution according to the manufacturer's protocol. The next day medium was changed to DMEM + 1% FBS. Virus supernatant was collected 48 h posttransfection, filtered through 0.45 µm filters, frozen in small aliquots on dry ice, and stored at −80°C. Twenty-four hours prior to infection, cells were seeded. The following day, the culture medium was aspirated and replaced with virus supernatant diluted 1:5 in the growth media. Medium was then changed into growth medium overnight. Twenty-four hours after infection, medium was changed into the selection medium (1.5 µg/ml puromycin containing growth medium). By the next 2 days, all non-infected cells were floating and presumably dead or dying. The infected cells were allowed to proliferate for 1 day, and then collected and re-seeded for the following experiments.

### Cancer cell line culture

MDA-MB 231, MDA-MB 468, Hs578T, HCC1569 and MCF-7 were obtained from the ATCC. HCC38, HCC70 and HCC1954 were kind gifts from Dr. Helene McMurray (University of Rochester, Rochester, NY, USA). MCF-10A was a generous gift from Dr. Patrica J. Simpson-Haidaris (University of Rochester, Rochester, NY, USA). The cell lines were grown using the recommended culture conditions (Neve et al, 2006).

### Cell viability assay

Cells were incubated with 500 nM Calcein-AM and 1 µM PI for 15 min, after which live cells and dead cells (represented by positivity of Calcein-AM and PI staining, respectively) were counted utilizing the adherent cell Celigo™ cytometer (Cyntellect, San Diego, California, United States).

### Immunoblotting

The cell culture samples were collected and lysed in RAPI buffer. Samples were resolved on SDS-PAGE gels and transferred to PVDF membranes (PerkinElmer Life Science, Wellesley, MA, USA). After being blocked in 5% bovine serum albumin in TBS containing 0.1% Tween 20, membranes were incubated with a primary antibody, followed by incubation with a HRP-conjugated secondary antibody (Santa Cruz Biotechnology). Membranes were visualized using Western Blotting Luminol Reagent (Santa Cruz Biotechnology).

### ROS measurement

Levels of intracellular stress of oxidation were determined by CM-H2DCFDA staining, according to the manufacturer's instructions. Briefly, cells were incubated with 1 µM CM-H2DCFDA in the phenol-red free medium in the dark for 30 min at 37°C, after which cells were washed once and the fluorescence intensity of cells was determined using adherent cell Celigo™ cytometer.

**The paper explained**

**PROBLEM**

Two of the most critical challenges in cancer research are to identify means of increasing the utility of existing therapeutic strategies and of discovering new vulnerabilities of cancer cells. Such challenges are most effectively achieved by the discovery of molecular mechanisms that address, and preferably integrate, these important goals.

**RESULTS**

We report that basal-like breast cancer (BLBC) cells use Cdc42 to inhibit function of the redox/Fyn/c-Cbl pathway (RFC), which normally enables small increases in oxidative status to cause enhanced degradation of c-Cbl target proteins. Restoration of normal RFC pathway function by genetic or pharmacological Cdc42 inhibition enabled us to harness the pro-oxidant effects of low µM concentrations of tamoxifen (TMX) – concentrations of TMX employed in trials on multiple tumour types – to suppress division and induce cell death in BLBC cells *in vitro* and to confer TMX sensitivity *in vivo* through oestrogen receptor-α-independent mechanisms. These studies also provide a pharmacological lead candidate Cdc42 inhibitor that confers TMX sensitivity *in vitro* and *in vivo*. In addition, we found that restoration of normal c-Cbl function via genetic inhibition of Cdc42 greatly inhibited mammosphere formation *in vitro* and tumour generation *in vivo*.

**IMPACT**

Our studies identify a single pathway that enhances the activity of TMX against BLBC cells and tumours and suppresses tumour initiation cell (TIC) function. The RFC pathway offers a rare example of a signalling pathway that appears integral to both resistance to therapeutic strategies and to the biology of TICs. Critically, our data demonstrates that it is not the ability of TMX to render cells more oxidized that was altered by restoring c-Cbl function, but the ability of cancer cells to escape the functional consequences of being more oxidized. These findings provide a new regulatory pathway that is subverted in cancer cells, a novel means of attacking TIC and non-TIC aspects of BLBCs and a lead molecule that confers sensitivity to low µM TMX. Our discoveries appear thus far to be unique in conferring sensitivity to a non-canonical mode of action of an established therapeutic agent, thus extending the potential use of TMX to a group of tumours that are otherwise non-responsive to this agent. The development of Cdc42 inhibitors suitable for use in the clinic could prove of considerable therapeutic value, as elevated levels of Cdc42 have been observed in multiple different types of cancers.

### Glutathione assay

The amount of GSSG and GSH was measured as described previously (Rahman et al, 2006). The ratio of GSSG/GSH was then calculated as an index of oxidative status.

### Cdc42/Rac1 activity assay

Measurement of activity of Cdc42 or Rac1 was conducted using G-LISA (Cytoskeleton, Inc, Denver, CO, USA) according to the instruction provided by the manufacturer.

### Immunoprecipitation

To examine activation of Fyn kinase, anti-Fyn polyclonal antibody was added into protein A/G agarose and then the protein A/G agarose was incubated with 0.75 mM discuccinimidylsuberate, crosslinking bound antibodies to protein A/G agarose. To examine ubiquitination of EGFR, anti-EGFR polycolonal antibody was added in A/G agarose. The mixtures were then added into cell lysates (300 µg of total protein in RIPA lysis buffer with protease and phosphatase inhibitors). Precipitates are resolved on an SDS–PAGE gel and then were subjected to Western blot analysis.

### Animal model

Breast cancer cell lines expressing firefly luciferase were suspended in 20 µL PBS and injected orthotopically into mammary fat pads of female NOD/SCID mice. Tumourigenicities of breast cancer cell lines were assessed in NOD/SCID mice by implantation with serially diluted numbers of cells treated with Cdc42, c-Cbl or scrambled shRNA constructs. Effects of systemic TMX administration on tumour growth in Cdc42 depleted breast cancer cells were examined by injection of 10^6^ MDA-MB 231 cells in the fat pads of female NOD/SCID mice monitoring the tumour growth. We initiated treatment with TMX [7.5 mg/60 days by subcutaneous implantation of releasing pellets (Innovative Research of America, Sarasota, FL, USA)] 20 days after mice were xenografted with a parallel comparison with a sham control group. To examine the effects of the combination of TMX + ML141, tumours were first established for 24 days prior to initiation of drug treatment. Animals were then treated with TMX (125 µg/day by intraperitoneal injection) and/or ML141 (1 mg/day by intraperitoneal injection) for 14 days. Six mice for each transplant were injected were euthanized when the tumours were approximately 2 cm in the largest diameter according to regulations for use of vertebrate animals in research. All procedures were approved by the University of Rochester Committee on Animal Resources.

### Bioluminescence detection

Tumour size was monitored using IVIS 100 Imaging station after intraperitoneal injection of 150 µg/g body weight d-luciferin. Normalized photon flux represents the ratio of the photon flux detected after inoculation to that detected before inoculation.

## Author contributions

HYC, YMY and BMS conducted all experiments and all were involved in data interpretation. MN supervised the research and data interpretation. The manuscript was written by HYC, BMS and MN.

## Notes

As a service to our authors and readers, this journal provides supporting information supplied by the authors. Such materials are peer reviewed and may be re-organized for online delivery, but are not copy-edited or typeset. Technical support issues arising from supporting information (other than missing files) should be addressed to the authors.
