# Supplementary material for: Inhibition of redox/Fyn/c-Cbl pathway function by Cdc42 controls tumour initiation capacity and tamoxifen sensitivity in basal-like breast cancer cells
Source: EMBO Mol Med. 2013 Apr 22;5(5):723–36. doi: 10.1002/emmm.201202140 (PMC3662315; doi:10.1002/emmm.201202140)
Supplement: Supplementary file 1 [file emmm0005-0723-sd1.pdf]

EMBO Molecular Medicine

Inhibition of redox/Fyn/c-Cbl pathway function by Cdc42 controls tumor initiation capacity and tamoxifen sensitivity in basal-like breast cancer cells

Hsing-Yu Chen, Yin Miranda Yang, Brett Stevens and Mark Noble

Corresponding author: Mark Noble, University of Rochester Medical Center

Review timeline:

Submission date:	10 October 2012
Editorial Decision:	11 December 2012
Revision received:	14 February 2013
Accepted:	05 March 2013

Transaction Report:

(Note: With the exception of the correction of typographical or spelling errors that could be a source of ambiguity, letters and reports are not edited. The original formatting of letters and referee reports may not be reflected in this compilation.)

Editor: Roberto Buccione

1st Editorial Decision 11 December 2012

Thank you for the submission of your manuscript to EMBO Molecular Medicine.

We are sorry for the delay in getting back to you with the Reviewers' evaluations. Unfortunately, in this case we experienced unusual difficulties in securing three appropriate reviewers in a timely manner. Since we cannot justify a further delay, we have made a decision based on two consistent evaluations.

You will see that while both reviewers are generally supportive of your work and underline its considerable potential interest, they also both raise a number of specific concerns that prevent us from considering publication at this time.

Reviewer 1 mentions two main points, which I feel to be important. S/he would like more insight into how cdc42 inhibition impacts on basal like breast cancer cell tumourigenicity and would also like to see confirmation that ML141 is specific for cdc42 at the higher doses used in your experiments compared to previously published work.

Reviewer 2, while also generally positive, raises numerous and specific concerns. Firstly, s/he mentions that your results need to be discussed in the light of previous work by the Cerione laboratory demonstrating the functional interaction between Cool-1 and cdc42 and its effect on Cbl. This is connected to Reviewer 1's comment on cdc42 inhibition. Reviewer 2 also suggests that your experiments should be extended to additional lines, more representative of the triple negative phenotype. Finally, s/he suggests that the claim that EGFR downregulation is driven by ubiquitination and lysosomal degradation should be better supported by experimental evidence. I

find these concerns to be relevant and should thus be appropriately addressed. Reviewer 2 also mentions other issues that need to be taken into consideration in a future version.

While publication of the paper cannot be considered at this stage, we would be pleased to consider a suitably revised submission, provided, however, that the Reviewers' concerns are fully addressed with additional experimental data where appropriate.

Please note that it is EMBO Molecular Medicine policy to allow a single round of revision only and that, therefore, acceptance or rejection of the manuscript will depend on the completeness of your responses included in the next, final version of the manuscript.

As you know, EMBO Molecular Medicine has a "scooping protection" policy, whereby similar findings that are published by others during review or revision are not a criterion for rejection. However, I do ask you to get in touch with us after three months if you have not completed your revision, to update us on the status. Please also contact us as soon as possible if similar work is published elsewhere.

I look forward to seeing a revised form of your manuscript as soon as possible.

***** Reviewer's comments *****

Referee #1 (Comments on Novelty/Model System):

The authors use 3 different basal like breast cancer cell lines in vitro and in vivo to show that inhibition of Cdc42 in combination with the well-established ER antagonist tamoxifen inhibits BLBC tumorigenicity. This study provides important insight into both the cellular and molecular mechanisms underlying the concerted action of a novel Cdc42 inhibitor and tamoxifen in targeting BLBC, and establishes a potential novel therapeutic strategy to treat a subtype of breast cancer that is particularly difficult to treat.

Referee #1 (General Remarks):

In the manuscript by Chen et al, using a variety of in vitro and in vivo approaches, the authors show that inhibition of Cdc42 sensitizes basal like breast cancer (BLBC) cells to the cytostatic and cytotoxic effects of the estrogen receptor antagonist, tamoxifen. This is a strong mechanistic study where the authors define both cellular and molecular mechanisms by which Cdc42 and tamoxifen work in concert to target BLBC cells. They show that the effects of Cdc42 inhibition are likely mediated by c-Cbl, which regulates turnover of a variety of proteins that contribute to tumor formation and progression, including EGFR. Furthermore, the authors show that Cdc42 regulates tumor initiating cell function. Importantly, this study demonstrates that a novel small molecule inhibitor ML141 that appears to be highly selective for Cdc42 and has efficacy against BLBC cells both in vitro and in vivo. ML141 doesn't impact the proliferation or survival of non-transformed MCF10A breast cells, suggesting that it is selective for BLBC cells. Development of Cdc42 selective inhibitors has been a particular challenge, and the results of this study will facilitate further development of Cdc42 inhibitors, which could be useful for treating a variety of different types of cancers. Lastly, the authors demonstrate that the non-canonical actions of tamoxifen, a well-established breast cancer therapeutic, can be harnessed to inhibit BLBC tumors, which is a breast cancer subtype that is particularly aggressive and resistant to other targeted therapies. I have just a few minor suggestions:

To better clarify the mechanism by which Cdc42 inhibition impacts BLBC tumorigenicity it would be helpful to confirm whether knockdown of cool-1 is affecting Cdc42 activity in the BLBC cell lines.

Page 9: What dose of TMX was used? The text is inconsistent with the dose shown in figure 3.

Although data are available from Surviladze et al, showing specificity of ML141 for Cdc42, it would be helpful to confirm ML141 specificity for Cdc42 at the significantly higher dose of 20 mM used in this study.

Referee #2 (General Remarks):

In this paper the authors examine the effects of Cdc42 knockdown or inhibition on the growth of breast cancer cell lines by tamoxifen. Tamoxifen has two modes of action. It inhibits the estrogen receptor, thus blocking estrogen dependent growth. However, tamoxifen also acts as an oxidant and at high concentrations can inhibit growth of cells independent of its action on the estrogen receptor. In previous work, these authors found that oxidants activated Fyn which in turn activates the ubiquitin ligase Cbl, leading to down regulation of Cbl substrates such as the platelet derived growth factor receptor. In this work, they investigate the role Cdc42 and Cbl may play in tamoxifen toxicity in cells that do not express estrogen receptor. They show that 1) tamoxifen at oxidizing doses does not result in Cbl phosphorylation despite Fyn activation; 2) knockdown of Cdc42 in MB231 cells results in increased Cbl phosphorylation and as a result EGFR downregulation; 3) pharmacologic inhibition of Cdc42 results in enhance toxicity of tamoxifen and is associated with EGFR down regulation; 4) that knockdown of Cbl prevents the enhanced toxicity of Cdc42 knockdown or pharmacologic inhibition when combine with tamoxifen; 5) that Cdc42 knockdown or pharmacologic inhibition in combination with tamoxifen results in a greater degree of tumor inhibition than either alone; and 6) Cdc42 knockdown reduces the ability of breast cancer cells to form mammospheres (as a measure of cancer initiating cells).

General Critique:

Overall the work proposes an interesting mechanism for using tamoxifen as an oxidant in combination with inhibition of Cdc42 in breast cancer cells. The work supports a model whereby Cdc42 prevents Cbl from targeting tyrosine kinases for degradation and the when Cdc42 is inhibited the activation of Cbl results in downregulation of these kinases and this ultimately inhibits cell growth when cells are treated with tamoxifen. The inhibition of Cbl by binding to Cdc42 is not novel (originally reported in Cell in 2003); however, this work highlights a therapeutic combination which may extend the use of tamoxifen to ER negative breast cancers.

The mechanism proposed requires some additional work. Based on the work by the Cerione lab, Cbl is constitutively associated with Cool-1. Cool-1 binds to the GTP bound form of Cdc42, thus sequestering Cbl and preventing Cbl from interacting with substrates such as the EGFR. Upon hydrolysis of GTP by Cdc42, the Cool-1-Cbl complex dissociates from Cdc42 and this frees Cbl to interact with, ubiquitinate, and downregulate its substrates. In the data presented here, the knock down of Cool-1 does not result in increased phosphorylation of Cbl has no effect on XYZ. Since Cbl is not known to directly interact with Cdc42, and since the model that has been published suggests the interaction is mediated by Cool-1, then these data require more work to explain how Cdc42, in the absence of Cool1, inhibits Cbl function. For example, does Cbl coIP with Cdc42 and if so does the knock down of Cool-1 affect the interaction of Cbl and Cdc42. If Cbl coIPs with Cdc42 in the absence of Cool-1, then what is mediating the interaction?

Also, does inhibition of Cdc42 with the compound ML141 disrupt the interaction between Cbl and Cdc42.

The title and paper overall emphasizes that tamoxifen + Cdc42 inhibition may be useful in basal breast cancer cells. In this study, they use three cell lines HCC38, MB231, and HCC 1954. The HCC38 and MB231 cells are triple negative breast cancers and classified as Basal B cells (basal with mesenchymal features) based on microarray. The HCC1954 cells are Her2 amplified tumor cells and the cells are classified as Basal A cells (basal with more epithelial features). Triple negative and Her2 amplified cancers are distinct from one another despite the array classification of all of these as basal. The data suggests that the combination of tamoxifen as an oxidant and inhibition of Cdc42 may be effective in a wide array of breast cancers. It would be helpful to test this in a panel of cells with more representative cell lines of that are triple negative, and more that are Her2 amplified to clarify this.

Specific critique:

1. Figure 1 and Figure 2 show composite densitometry bar graphs of phospho-Cbl and EGFR levels but the blots are shown as supplemental data. The blots are the primary data and so should be shown in the main figure and not as a supplement.

2. In figure 2A there is a ~3 fold increase in Cbl phosphorylation when Cdc42 is knocked down which is increased further by treatment with tamoxifen. However, in the blots shown in supplementary figures 2, 4 and 5 there is no blot that has the phosphorylation levels of Cbl that would correspond to the figure shown in Figure 2A. That is, Supplementary figure 2 shows p-c-Cbl levels in cells without Cdc42 knock down and supplementary figures 4 and 5 only show p-c-Cbl in cells with Cdc42 knocked down. The authors should show a blot that would allow the reader to see the results as quantified in Figure 2A.

3. Based on the model, the downregulation of EGFR by tamoxifen with the loss of Cdc42 should be driven by ubiquitination and lysosomal degradation of the EGFR. This should be demonstrated.

4. In supplementary figure 5, the phosphorylation of Cbl is essentially absent when Cool-1 is knocked down. As mentioned above, Cool-1 is thought to be an adaptor between Cbl and Cdc42. Since binding of Cbl to Cdc42 inhibits activity, it would be predicted that in the absence of Cool-1, Cbl should be released from Cdc42 and phosphorylated. This requires a discussion.

5. In Figure 3C, D and F, there are no blots that demonstrate the Cdc42 knockdown. As I read the methods, the shRNA knockdowns were done using lentiviral vectors but the method does not imply that stable clones or pools were selected and maintained. If the knockdowns were done separately for each experiment, then the blots should be shown. If the knockdown cells were the ones used in figure 2, this should be stated. A similar comment applies to figure 4, 5 and 7.

1st Revision - authors' response 14 February 2013

February 11, 2013

The Editors
EMBO Molecular Medicine

Dear Editors:

Thank you for forwarding to us the thoughtful reviews and enthusiastic response to our manuscript. We were very pleased to see that both Reviewers were generally supportive of our work and underlined its considerable potential interest and potential therapeutic relevance, and that you were interested in seeing a revised manuscript.

We have conducted multiple additional experiments, added data and modified the manuscript to address the questions raised in the review process, and provide a point-by-point response to these concerns. We apologize for the length of this reply, which is a reflection of the seriousness with which we have taken the referee's questions. Happily, the requested experiments have all supported the results of the original manuscript.

Reviewer 1

We very much appreciate the multiple kind comments of Reviewer 1, who thought our work provided " *important insight into both the cellular and molecular mechanisms underlying the concerted action of a novel Cdc42inhibitor and tamoxifen in targeting BLBC, and establishes a potential novel therapeutic strategy to treat a subtype of breast cancer that is particularly difficult to treat."* The reviewer went on to say that "*this is a strong mechanistic study"*and that "*the results of this study will facilitate further development of Cdc42 inhibitors, which could be useful for treating a variety of different types of cancers. Lastly, the authors demonstrate that the non-canonical actions of tamoxifen, a well-established breast cancer therapeutic, can be harnessed to inhibit BLBC tumors, which is a breast cancer subtype that is particularly aggressive and resistant to other targeted therapies"*.

Reviewer 1 made three requests, all of which we have addressed as follows:

1. To better clarify the mechanism by which Cdc42 inhibition impacts BLBC tumorigenicity it would be helpful to confirm whether knockdown of cool-1 is affecting Cdc42 activity in the BLBC cell lines.

We agree that defining whether Cool-1 knockdown affects Cdc42 activity in BLBCs would help in understanding possible interactions between Cool-1 and Cdc42 in these cells and thereby clarify whether Cdc42 prevents c-Cbl function in a-Cool-1 dependent manner to regulate tumor initiation by BLBCs.

To answer this question, we first generated Cool-1 knockdown MDA-MB 231 cells by lentiviral infection and measured Cdc42 activity by Cdc42-specific G-LISA. In the MDA-MB 231 cells, Cool-1 knockdown did not alter Cdc42 activity compared to the scrambled controls (as shown in **Figure S3A** in the revised

manuscript), strongly suggesting that Cdc42 function is not controlled by Cool-1 in BLBCs and offering a possible explanation as to why Cool-1 knockdown did not restore c-Cbl function (as shown in **Figure S3** in the revised manuscript) and why Cool-1 knockdown did not increase sensitivity of BLBC cells to TMX (as shown in **Figure S8** in the revised manuscript).

Thus, it appears that the view that Cdc42 and Cool-1 necessarily work together to inhibit c-Cbl is incorrect, consistent with our initial observations.

2. Page 9: What dose of TMX was used? The text is inconsistent with the dose shown in figure 3.

We thank the reviewer for pointing out the typos that might be the cause of the misunderstandings of the concentrations used in this study. The concentrations of TMX, ML141 and GGTI-298 were in the low μM range as mentioned in the figures and legends. We have added the corrected concentrations of these agents in the revised manuscript.

3. Although data are available from Surviladze et al, showing specificity of ML141 for Cdc42, it would be helpful to confirm ML141 specificity for Cdc42 at the significantly higher dose of 20 mM used in this study.

We highly agree with the important concern that the high dosages of ML141 might create off-target effects by which enhancement of the TMX killing in BLBC cells we observed in this study might not be relevant to Cdc42 inhibition. First we would like to point out that in this study we continuously used 20μM ML141 in all *in vitro* assays as discussed in the answer to reviewer1's second question. Nonetheless, 20μM ML141 is still 2 times higher than the concentration the Surviladze group used in 3T3 cells in the previous study. To confirm the specificity of this concentration of ML141 in BLBCs, we measured the activation of Cdc42 and Rac1 using G-LISA in MDA-MB 231 cells treated with increasing concentrations of ML141. We found that 20μM ML141 selectively inhibited activation of Cdc42 by ~50% (but not Rac1) and that the lower concentrations (5μM or 10μM) of ML141 were not able to reduce the activity of either Cdc42 or Rac1 in BLBCs (as shown in **Figure S4** in the revised manuscript). This dose-dependence of Cdc42 inhibition agrees with our observation in which BLBCs treated with 20μM ML141 became more sensitive to TMX than un-dosed controls but lower doses of ML141 failed to increase vulnerability of these BLBC cells to TMX.

Reviewer 2

We thank Reviewer 2 for his/her generally positive comments and constructive suggestions.

1. Overall the work proposes an interesting mechanism for using tamoxifen as an oxidant in combination with inhibition of Cdc42 in breast cancer cells. The work supports a model whereby Cdc42 prevents Cbl from targeting tyrosine kinases for degradation and the when Cdc42 is inhibited the activation of Cbl results in downregulation of these kinases and this ultimately inhibits cell growth when cells are treated with tamoxifen. The inhibition of Cbl by binding to Cdc42 is not novel (originally reported in Cell in 2003); however, this work highlights a therapeutic combination which may extend the use of tamoxifen to ER negative breast cancers.

The mechanism proposed requires some additional work. Based on the work by the Cerione lab, Cbl is constitutively associated with Cool-1. Cool-1 binds to the GTP bound form of Cdc42, thus sequestering Cbl and preventing Cbl from interacting with substrates such as the EGFR. Upon hydrolysis of GTP by Cdc42, the Cool-1-Cbl complex dissociates from Cdc42 and this frees Cbl to interact with, ubiquitinate, and downregulate its substrates. In the data presented here, the knock down of Cool-1 does not result in increased phosphorylation of Cbl has no effect on XYZ. Since Cbl is not known to directly interact with Cdc42, and since the model that has been published suggests the interaction is mediated by Cool-1, then these data require more work to explain how Cdc42, in the absence of Cool-1, inhibits Cbl function. For example, does CblcoIP with Cdc42 and if so does the knock down of Cool-1 affect the interaction of Cbl and Cdc42. If Cbl co-IPs with Cdc42 in the absence of Cool-1, then what is mediating the interaction?

The insightful studies of the Cerione laboratory have played a critical role in our thinking about c-Cbl inhibition. It is important to remember, however, that these studies were carried out using over-expression experiments and did not demonstrate that interactions of this nature occur in human tumor cells. We are all aware that the protein-protein interactions that can be detected in the context of over-expression can be much more difficult to detect in the context of expression levels that occur in normal cells or cell lines. Thus, it is critical to conduct other kinds of experimentations to test hypotheses regarding the importance of particular proteins in a proposed regulatory network.

Several lines of evidence indicate that Cool-1 does not appear to be the essential protein regulating c-Cbl function in BLBCs:

(i) First, as shown in the initial manuscript, Cool-1 knockdown in MDA-MB 231 cells did not restore endogenous and TMX-induced phosphorylation of c-Cbl (also shown in **Figure S3C** in the revised manuscript) and did not enhance sensitivity to TMX *in vitro* (also shown in **Figure S8** in the revised manuscript).

(ii) Second, as discussed in the response to referee1's concerns, Cool-1 knockdown did not affect Cdc42 activity in MDA-MB 231 cells, suggesting the regulatory role of Cool-1 on Cdc42 is not detectable in theses cells (also shown in **Figure S3A** in the revised manuscript).

(iii)Third, when we performed the c-Cbl co-IP experiment as referee 2 suggested, it did not reveal a detectable direct interaction between c-Cbl, Cool-1 and Cdc42. This does not mean the interactions do not occur, for it may be that at normal levels of protein expression they are too evanescent to be captured in a co-immunoprecipitation experiment. Nonetheless, in light of points (i) and (ii), a cautious interpretation of the data would be that the effects of Cdc42 on c-Cbl function in these cells are Cool-1 independent.

Although above results support the interpretation that Cdc42-mediated suppression of c-Cbl is not mediated via Cool-1, they do not change any of the evidence regarding the importance of the restoration of activity of c-Cbl, the inhibition of its regulation by the redox/Fyn/c-Cbl (RFC) pathway in BLBC cells, the ability of restoring RFC pathway activity to confer sensitivity to TMX via ER-independent mechanisms or the other novel findings in our manuscript.

We have tried to err on the side of caution in the interpretations we make in this regard. Co-immunoprecipitation studies in cells that are not modified to overexpress proteins of interest may not reveal interactions that do occur but are evanescent. However, what does appear to be an appropriate interpretation is that Cdc42-mediated inhibition does not require Cool-1.

Like the reviewer, we also are very interested in the details of this pathway. We therefore also examined Cdc42-interacting protein 4 (CIP4), which is overexpressed in more invasive breast cancer cells (Pichot et al, 2010, Cancer Res. 70, 8347-8356) has been proposed to regulate c-Cbl function by sequestration (Dombrosky-Ferlan et al., 2003, Blood 101, 2804-2809).

Analysis of whether CIP4 knockdown restores c-Cbl function, as tested by reductions in EGFR levels yielded intriguing outcomes. We found that MDA-MB 231 cells with reduced CIP4 expression had lower levels of EGFR compared to scrambled controls and that a 4 hour exposure of these cells to 10μM TMX further caused reductions in EGFR levels. Thus, CIP4 knockdown also appeared to restore c-Cbl function, raising the possibility that CIP4 could be a potential mediator by which Cdc42 regulates c-Cbl function in BLBCs. This might not even be due to physical sequestration, however, as we did not detect any interaction between c-Cbl and CIP4/Cdc42 in c-Cbl co-IP experiments. We have added the idea of Cdc42/CIP4 being a

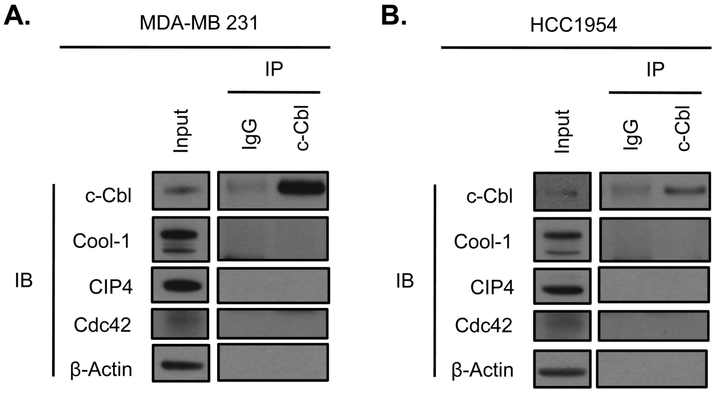

potential component of the mechanism by which Cdc42 regulates c-Cbl function in BLBCs in the discussion section in the revised manuscript and have included the relevant data in **Figure S13**. We also show below the results of immunoprecipitation experiments conducted in MDA-MB-231 cells and HCC1954 cells. In both cases, immunoprecipitation with anti-c-Cbl antibodies does not pull down detectable Cool-1, CIP4, or Cdc42. This is not a problem with our immunoprecipitation experiments, as separate analyses conducted on glioblastoma cells do reveal the existence of complexes between c-Cbl and Cool-1 in similar experiments. Similarly, in the studies on glioblastoma cells, we find that the reciprocal data as presented in the present study, which is that c-Cbl is inhibited by Cool-1 but not by Cdc42. This data is not shown, as these studies are focused on a completely different type of cancer that is not characterized by Cdc42 overexpression. While it is of interest that different types of cancers may use different proteins to inhibit c-Cbl activity, it needs to be emphasized that the present studies are focused on a very different question than the mechanisms of such inhibition.

Despite our shared interest in understanding the mechanism by which Cdc42 inhibits c-Cbl, we hope the reviewer will agree, however, that the dissection of the precise mechanism by which Cdc42 inhibits c-Cbl is, at most, a secondary aspect of this paper and that the outcomes of these experiments do not alter the molecular medicine-related importance of our studies. The mechanistic dissection of how Cdc42 is regulated does not affect the interpretation that Cdc42 regulates c-Cbl activation, an interpretation supported by multiple lines of evidence in our studies, both genetic and pharmacological. The genetic outcomes are as predicted by previous studies, while the pharmacological experimentation adds important new aspects to such work. The details of how this interaction occurs do not change the outcome of the experiments

demonstrating that genetic and pharmacological inhibition of Cdc42 restore redox-mediated activation of c-Cbl and confers sensitivity to TMX. Had we seen the interactions predicted by the paper from the Cerione laboratory, this would not have altered any of our novel observations. Not finding these predicted interactions suggests that there likely is more to be discovered about how Cdc42 modulates c-Cbl activity, but also does not alter the observations that such modulation offers a promising approach for beneficially modulating the biology of BLBC cells.

The above results could be interpreted in support of an unknown non-canonical pathway occurring in BLBC cells to mediate Cdc42 in suppression of c-Cbl function, an interpretation we have included in the discussion together with the results of our experiments on Cip4.

2. Also, does inhibition of Cdc42 with the compound ML141 disrupt the interaction between Cbl and Cdc42.

We agree with the rationale for asking whether ML141 exposure disrupts the interaction between c-Cbl and Cdc42, and we examined this possibility while considering other evidence for such interactions. However, as no interaction between c-Cbl and Cdc42 could be seen in BLBC cells (as discussed in the response to referee 2's first comment), there was also no ability to detect interruption of these two proteins by exposure to ML141.

3. The title and paper overall emphasizes that tamoxifen + Cdc42 inhibition may be useful in basal breast cancer cells. In this study, they use three cell lines HCC38, MB231, and HCC 1954. The HCC38 and MB231 cells are triple negative breast cancers and classified as Basal B cells (basal with mesenchymal features) based on microarray. The HCC1954 cells are Her2 amplified tumor cells and the cells are classified as Basal A cells (basal with more epithelial features). Triple negative and Her2 amplified cancers are distinct from one another despite the array classification of all of these as basal. The data suggests that the combination of tamoxifen as an oxidant and inhibition of Cdc42 may be effective in a wide array of breast cancers. It would be helpful to test this in a panel of cells with more representative cell lines of that are triple negative, and more that are Her2 amplified to clarify this.

We are grateful to the reviewer for making this request. As Reviewer 2 points out, it is important to consider whether TMX plus Cdc42 inhibition is effective in targeting all three distinct subtypes of BLBCs. We therefore assembled a panel of seven cell lines with different properties. These included another basal-B cell line (Hs578T), two basal-A cell lines (MDA-MB 468 and HCC70) and another basal-A cell line with HER2 amplification (HCC1569). We examined all these cells in *in vitro* assays to test whether Cdc42 inhibition with ML141 renders all these cells vulnerable to TMX.

We found that exposure to 20μM ML141 sensitized all three different types of BLBCs to 10μM TMX (as shown in **Figure 3A** in the revised manuscript). ML141 alone did not cause significant reductions in cell number in BLBC cells, but ML141 enabled TMX to greatly reduce cell number of all three types of BLBC cells to less than 30% of the cell number seen in un-dosed controls or cells treated with single agents. Thus, ML141 enhanced TMX sensitivity in all of these populations, with no striking differences in outcome. The consistently identical outcome in these results argues that the effects of ML141 and Cdc42 inhibition were not restricted to any particular subset of BLBCs.

Addition of more cell lines to the analysis also enabled us to make an additional discovery that we thought intriguing enough to include in the supplementary information, even though at the moment it is a nuance.

Inhibition of GGTase I – which prenylates Cdc42 and is thought to tightly regulate its function – also made BLBC cells more sensitive to TMX (as shown in **Figure S6** in the revised manuscript). In cells treated with 5μM GGTI-298 (a specific inhibitor for GGTase I used and shown in the original manuscript), 10μM TMX caused generally marked reductions in cell numbers. Interestingly, HCC70 (a basal-A type cell line) appeared to be resistant to the combination of GGTI-298 + TMX although it was sensitive to the combination of ML141 + TMX. We included this finding in the revised manuscript as the different outcomes between these cell lines may provide a unique tool for laboratories interested in the nuanced differences between regulation of GGTase I and Cdc42 as therapeutic targets.

4. Figure 1 and Figure 2 show composite densitometry bar graphs of phospho-Cbl and EGFR levels but the blots are shown as supplemental data. The blots are the primary data and so should be shown in the main figure and not as a supplement.

We are delighted to move the blots from the supplement to the main figures and have moved all the blots from the supplement to the main **Figure 1** and **Figure 2** in the revised manuscript.

5. In figure 2A there is a ~3 fold increase in Cbl phosphorylation when Cdc42 is knocked down which is increased further by treatment with tamoxifen. However, in the blots shown in supplementary figures 2, 4 and 5 there is no blot that has the phosphorylation levels of Cbl that would correspond to the figure shown in Figure 2A. That is, Supplementary figure 2 shows p-c-Cbl levels in cells without Cdc42 knock down and supplementary figures 4 and 5 only show p-c-Cbl in cells with Cdc42 knocked down. The authors should show a blot that would allow the reader to see the results as quantified in Figure 2A.

We thank the reviewer for this careful examination of the data and for requesting the blot demonstrating that Cdc42 knockdown alone revealed a ~3 fold increase in phosphorylation of c-Cbl and that exposing these cells to TMX further increased c-Cbl phosphorylation. We have added the blot for **Figure 2A** in this regard and also added an additional blot showing Cdc42 indeed was reduced using shRNAs in the revised manuscript.

6. Based on the model, the downregulation of EGFR by tamoxifen with the loss of Cdc42 should be driven by ubiquitination and lysosomal degradation of the EGFR. This should be demonstrated.

We agree with the importance of demonstrating c-Cbl's relevance to EGFR degradation-induced by Cdc42 inhibition and TMX exposure. We showed in the original manuscript that secondary c-Cbl knockdown in Cdc42 knockdown cells suppressed TMX-induced reductions in levels of EGFR observed in Cdc42 knockdown cells, suggesting c-Cbl plays a key role in this regulation (as shown in **Figure 2D** in the revised manuscript).

To further confirm c-Cbl's involvement as a known E3 ubiquitin ligase that enhances the degradation of EGFR by increases in ubiquitination of EGFR and in lysosomal degradation, we conducted the experiments requested by the reviewer.

In MDA-MB 231 cells expressing Cdc42 shRNAs, ubiquitination of EGFR was increased after 12 hours of TMX exposure compared to Cdc42 knockdown cells or to control (Scr construct) cells exposed to either vehicle or TMX (as shown in **Figure 2E** in the revised manuscript). Similarly, MDA-MB 231 or HCC1954

cells treated for 24 hours with 20μM ML141 revealed a slight increase in ubiquitination of EGFR. Moreover, cells exposed to the combination of ML141 plus TMX showed even higher levels of EGFR ubiquitination (as shown in **Figure S5** in the revised manuscript).

The finding further confirms our and other's observations in which Cdc42inhibition restores c-Cbl function and thereby enhances c-Cbl mediated EGFR degradation (at least partially through increases in ubiquitination of this receptor). To further confirm this c-Cbl-mediated EGFR degradation was via lysosomes, MDA-MB 231 cells with reduced Cdc42 expression were co-exposed to TMX plus the lysosomal inhibitor ammonium chloride (NH_4Cl) for 12 hours. As predicted as a consequence of lysosomal inhibition, we found that exposure of Cdc42 knockdown cells to 20mM NH_4Cl and to 10μM TMX failed to reduce levels of EGFR protein (as shown in **Figure 2F** in the revised manuscript). This finding suggested that Cdc42 inhibition plus TMX in BLBCs activated c-Cbl function, leading to increases in lysosomal-related EGFR degradation. All of these findings have been added in the revised manuscript.

7. In supplementary figure 5, the phosphorylation of Cbl is essentially absent when Cool-1 is knocked down. As mentioned above, Cool-1 is thought to be an adaptor between Cbl and Cdc42. Since binding of Cbl to Cdc42 inhibits activity, it would be predicted that in the absence of Cool-1, Cbl should be released from Cdc42 and phosphorylated. This requires a discussion.

As mentioned in the response to referee 2's earlier comments, in BLBC cells the Cdc42-mediated regulation of c-Cbl function did not appear to be due to physical sequestration by Cool-1 and/or Cdc42 (or even by CIP4, as discussed earlier). We have commented in this regard in the discussion section in the revised manuscript. Elucidation of how the unknown, and apparently non-canonical, pathway mediates Cdc42 to regulate c-Cbl will be an interesting topic for further investigation but, as noted earlier, does not alter any of the primary observations reported in our studies or the interpretations of these findings.

8. In Figure 3C, D and F, there are no blots that demonstrate the Cdc42 knockdown. As I read the methods, the shRNA knockdowns were done using lentiviral vectors but the method does not imply that stable clones or pools were selected and maintained. If the knockdowns were done separately for each experiment, then the blots should be shown. If the knockdown cells were the ones used in figure 2, this should be stated. A similar comment applies to figure 4, 5 and 7.

We apologize that we assumed readers would assume (correctly) that Cdc42 knockdown cells can be generated using our lentiviral method by just once showing Figure 2A in the initial manuscript, but we are happy to agree with referee 2 that it is critical to demonstrate the efficiency of reduction in Cdc42 levels in each experimental setting. As indicated in Materials and Methods, these experiments were not conducted by generating a single stable clone and also did not keep using the same mixed cell pool. Instead, we freshly prepared Cdc42 shRNA virus and infected BLBC cells overnight, after which puromycin was used to positive select out cells bearing Cdc42 shRNA vectors. In this way, the outcomes of our experiments were successfully repeated many times during the course of these experiments.

To address this request, we have added Cdc42 blots for **Figure 2**, **Figure 3**, **Figure 5** and **Figure 7** in the revised manuscript.

Additional information added to the manuscript

We also have added two further sets of data, which provide further support for our observations. These are as follows:

1. We added further supporting evidence for the hypothesis that Cdc42 regulates the tumor initiation properties of BLBCs by applying one of the widely used experimental paradigms in the literature on tumor initiating cells, in which cells are pre-treated with the drug of interest prior to transplantation to determine if there is a change in tumor generation (as shown in **Figure S11** in the revised manuscript). In MDA-MB 231 cells pre-exposed for 40 hours to DMSO or 20µM ML141 (a concentration showing no apparent effects on BLBC cell number/survival, as shown in **Fig 3A** in the revised manuscript), mice transplanted with 10,000 ML141 pre-treated cells failed to form tumors at 20 days post-transplantation, a time point when 4 out 5 animals transplanted with DMSO-pretreated cells generated tumors. Even at 40 days after implantation, only 2 out of 5 animals implanted with ML141 pretreated cells generated tumors and the sizes of these tumors were significantly smaller than those from the DMSO pretreatment group.

2. We also added *in vivo* evidence to further demonstrate that the enhanced survival of TMX-treated animals bearing Cdc42 knockdown cells was c-Cbl dependent (as shown in **Figure S9** in the revised manuscript). TMX treatment *in vivo* failed to extend the survival time of animals implanted with cells expressing with both c-Cbl and Cdc42 shRNAs. The median survival time of mice transplanted with cells expressing both c-Cbl and Cdc42 shRNAs was 96 days, and was unchanged (98 days) when such animals were treated with TMX.

We very much hope that with the addition of the extensive experimentation conducted in response to the constructive requests of the reviewers, and our attention to addressing other requests for clarification, that this manuscript will now be considered suitable for publication in *EMBO Molecular Medicine*.

Accepted 05 March 2013

Please find enclosed the final reports on your manuscript. We are pleased to inform you that your manuscript is accepted for publication and is now being sent to our publisher to be included in the next available issue of EMBO Molecular Medicine.

Congratulations on your interesting work,

***** Reviewer's comments *****

Referee #1 (Comments on Novelty/Model System):

The authors have adequately addressed my concerns with the addition of several new experiments that clarify and strengthen the conclusions of the manuscript.

Referee #1 (General Remarks):

The authors have adequately addressed my comments, and I believe the manuscript is suitable for publication.

Referee #2 (Comments on Novelty/Model System):

The work provides a novel approach to the treatment of triple negative and HER2 positive breast cancers with tamoxifen and inhibition of Cdc42 and should be of interest to a wide audience.

Referee #2 (General Remarks):

The authors have addressed the critique and the paper should be published
