# Supplementary material for: Inhibition of redox/Fyn/c-Cbl pathway function by Cdc42 controls tumour initiation capacity and tamoxifen sensitivity in basal-like breast cancer cells
Source: EMBO Mol Med. 2013 Apr 22;5(5):723–36. doi: 10.1002/emmm.201202140 (PMC3662315; doi:10.1002/emmm.201202140)
Supplement: Supplementary file 2 [file emmm0005-0723-sd2.pdf]

Table of contents

Supporting information Fig 1. Schematic summary of the redox/Fyn/c-Cbl pathway.
Supporting information Fig 2. TMX exposure renders BLBC cells more oxidized.
Supporting information Fig 3. Cool-1 knockdown does not restore c-Cbl phosphorylation in BLBC cells
Supporting information Fig 4. ML141 selectively inhibits Cdc42 activity in BLBCs.
Supporting information Fig 5. ML141 enables TMX to cause reductions in EGFR levels in BLBCs.
Supporting information Fig 6. Inhibition of GGTase I increases TMX sensitivity in BLBC cells.
Supporting information Fig 7. Inhibition of Cdc42 increases TMX sensitivity in BLBC cells.
Supporting information Fig 8. Cool-1 knockdown fails to increase sensitivity of BLBCs to TMX.
Supporting information Fig 9. Reducing c-Cbl expression suppresses sensitivity of Cdc42 knockdown BLBC cells to TMX sensitivity *in vivo*.
Supporting information Fig 10. ML141+TMX suppresses tumor growth of BLBC cells *in vivo*.
Supporting information Fig 11. ML141 pretreatment decreases frequency of tumor formation of BLBCs.
Supporting information Fig 12. Cdc42 knockdown suppresses tumor growth of BLBCs *in vivo*.
Supporting information Fig 13. CIP4 knockdown enables TMX to cause reductions in EGFR levels.
Supporting information Fig 14. Expression of ERs is not altered upon knockdown or inhibition of Cdc42.

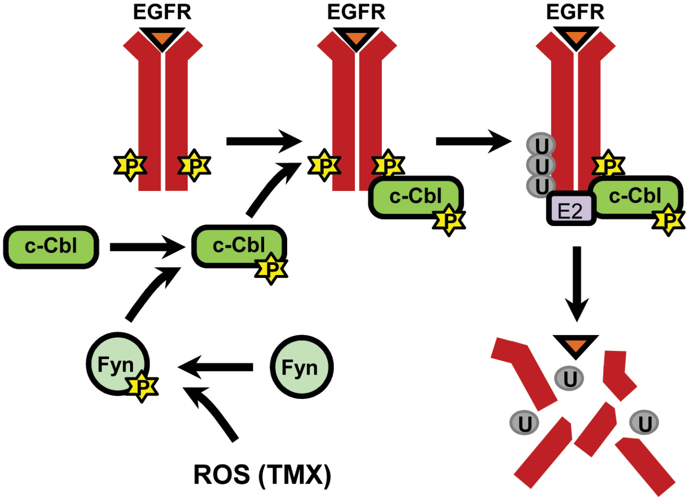

Supporting information Fig 1. Schematic summary of the redox/Fyn/c-Cbl pathway. Slight oxidative changes (such as induced by exposure to TMX) cause activation of Fyn kinase, leading to phosphorylation of c-Cbl E3 ubiquitin ligase and ubiquitylation and enhanced degradation of its target proteins, such EGFR.

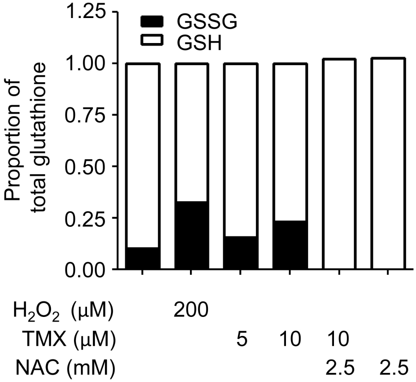

Supporting information Fig 2. TMX exposure renders BLBC cells more oxidized. MDA-MB 231 cells were exposed to hydrogen peroxide or to increasing dosages of TMX in the presence or in the absence of 2.5mM NAC, after which oxidized and total glutathione were measured.

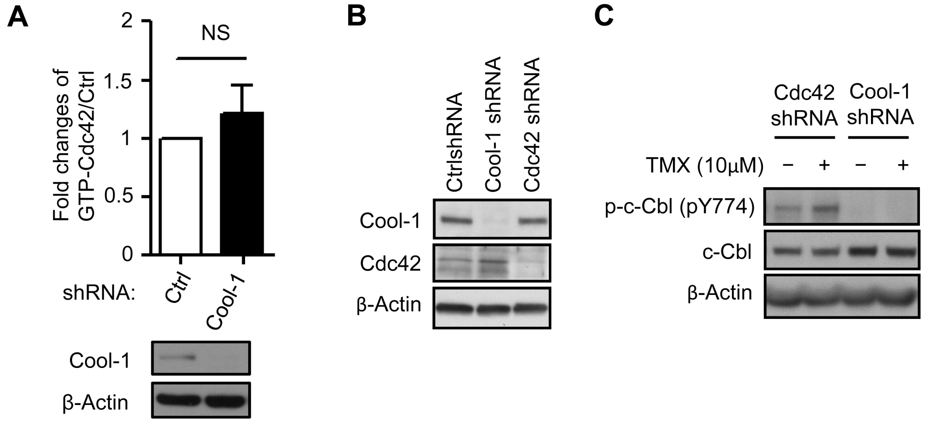

Supporting information Fig 3. Cool-1 knockdown does not restore c-Cbl phosphorylation in BLBC cells. (A) Cdc42 activity in MDA-MB 231 cells bearing scrambled or Cool-1 shRNAs was measure by G-LISA. (B) MDA-MB 231 cells were lentivirally infected for expression of Cool-1 or Cdc42 shRNAs, after which Cool-1 and Cdc42 expression were determined by immunoblotting of cells. (C) These genetically modified MDA-MB 231 cells were exposed to 10μM TMX for 1 hour. Activation of c-Cbl, represented by its phosphorylation at the Tyrosine residue 774, was examined through immunoblotting.

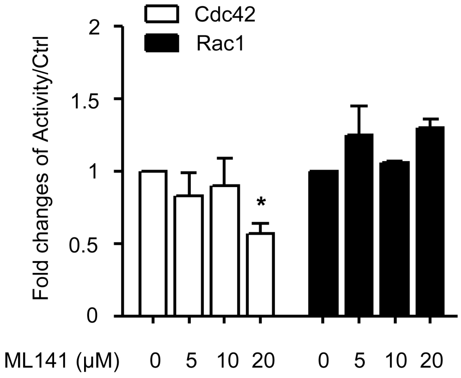

Supporting information Fig 4. ML141 selectively inhibits Cdc42 activity in BLBCs. The activity of Cdc42 and Rac1 in MDA-MB 231 cells treated with increasing concentrations of ML141 for 24 hours was measured by G-LISA.

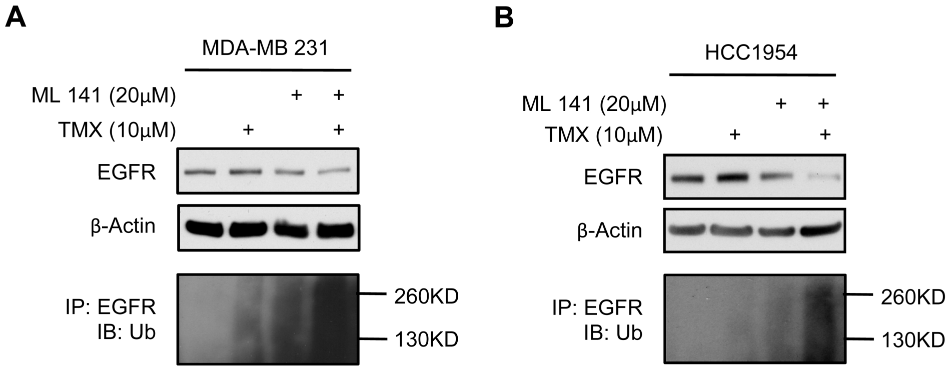

Supporting information Fig 5. ML141 enables TMX to cause reductions in EGFR levels in BLBCs. MDA-MB 231 cells and HCC1954 cells were pretreated with 20μM ML141 for 12 hours and then exposed to 10μM TMX for 12 hours, after which cells were harvested and examined for ubiquitination of EGFR and total EGFR expression by Western blotting.

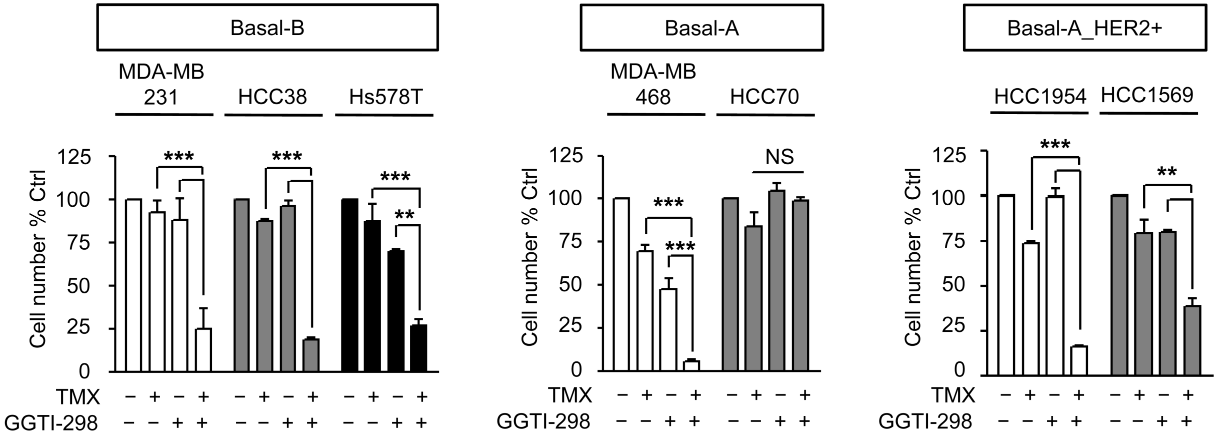

Supporting information Fig 6. Inhibition of GGTase I increases TMX sensitivity in BLBC cells. BLBC cells were exposed to 10μM TMX plus 5μM GGTI-298 for 48 hours. Viable cell number was measured by Calcein-AM+ analyses on the Celigo™. Numbers represent percentages as compared to un-treated controls. Data represented Mean±SEM (N≧3). Student's t-test was performed. **, $P < 0.01$; ***, $P < 0.001$.

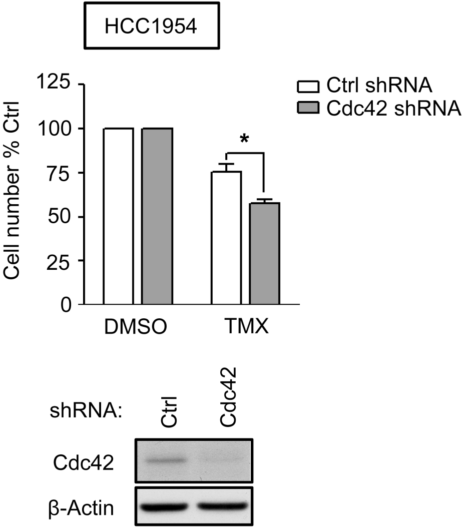

Supporting information Fig 7. Inhibition of Cdc42 increases TMX sensitivity in BLBC cells. HCC1954 cells expressing shRNAs for Cdc42 were exposed to 10μM TMX for 48 hours. Viable cell number was measured by Calcein-AM+ analyses on a Celigo™. Numbers represent percentages as compared to un-treated controls. Data represented Mean±SEM (N≧3). Student's t-test was performed. *, $P < 0.05$.

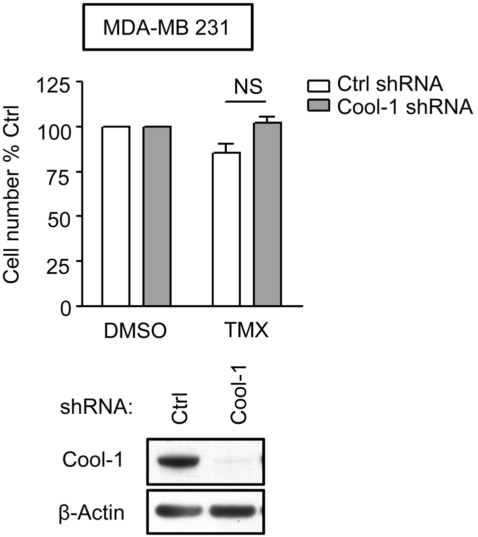

Supporting information Fig 8. Cool-1 knockdown fails to increase sensitivity of BLBCs to TMX. MDA-MB 231 cells expressing shRNA for Cool-1 were exposed to 10μM TMX for 48 hours. Viable cell number was measured by Calcein-AM+ analyses on a Celigo™. Numbers represent percentages as compared to un-treated controls. Data represented Mean±SEM (N≧3). Student's t-test was performed.

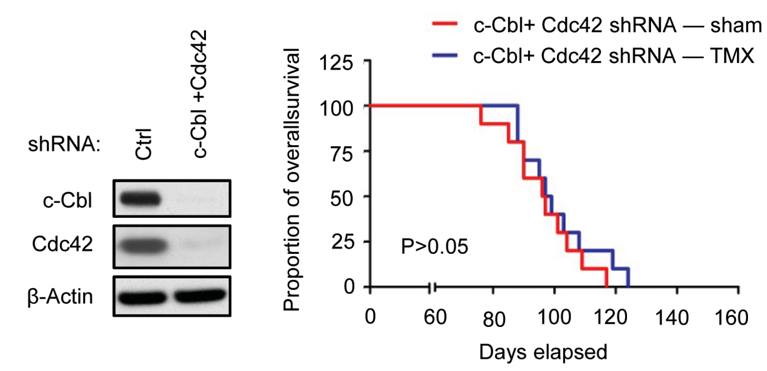

Supporting information Fig 9. Reducing c-Cbl expression suppresses sensitivity of Cdc42 knockdown BLBC cells to TMX sensitivity *in vivo*. For each xenograft, 1,000,000 cells were transplanted into mammary fat pad of a female NOD/SCID mouse, and at day 20 after transplantation, a TMX releasing pellet (7.5mg/60days) was subcutaneously implanted. The survival of mice with breast tumors was recorded and analyzed by Mantel-Cox test. The p value refers to the comparison among all groups.

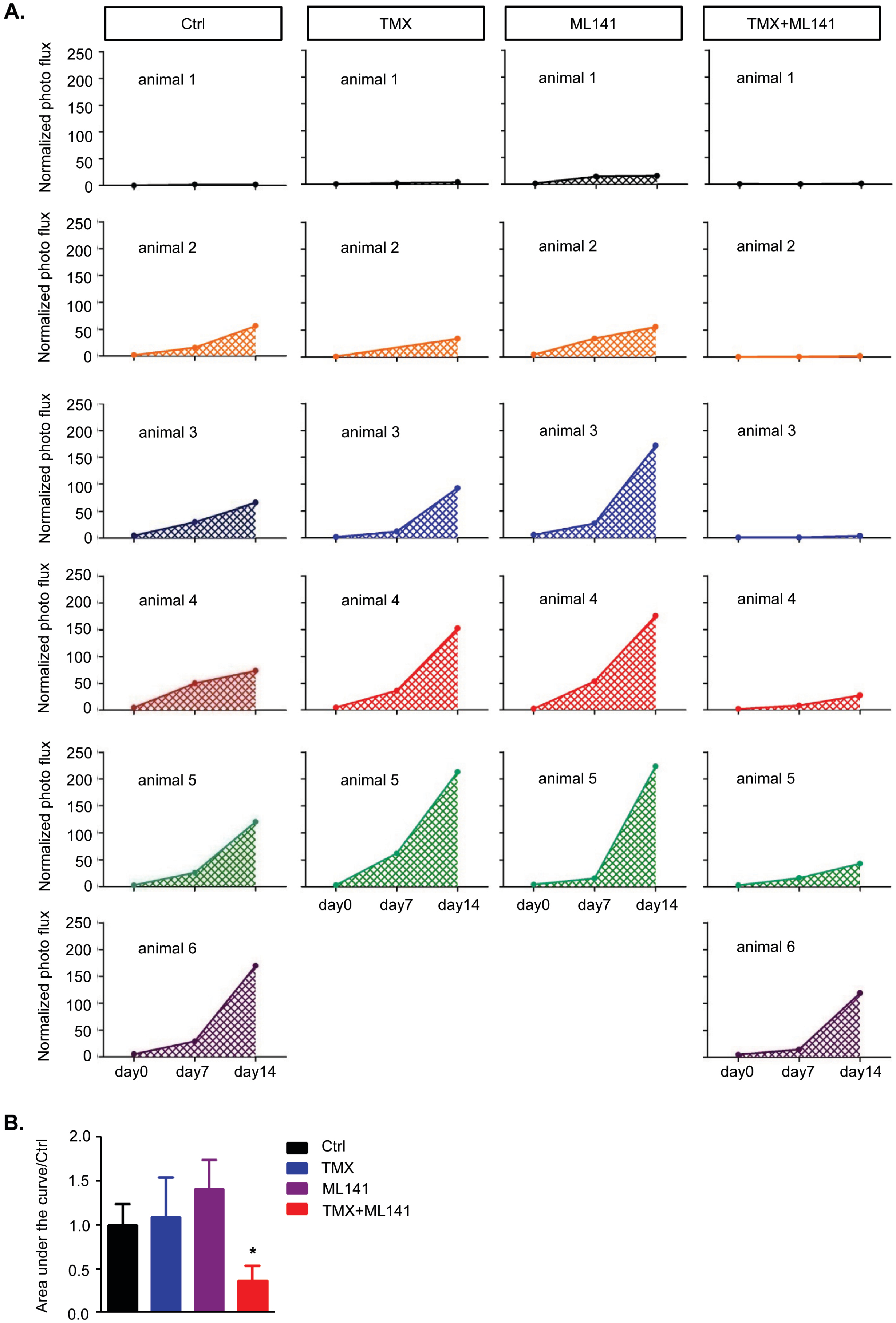

Supporting information Fig 10. ML141+TMX suppresses tumor growth of BLBC cells *in vivo*. (A) Tumor size of each animal implanted with 1,000,000 MDA-MB 231 cells was monitored and displayed quantitatively. (B) The area under each line was measured and one-tailed student's t-test was performed to compare to the control group.

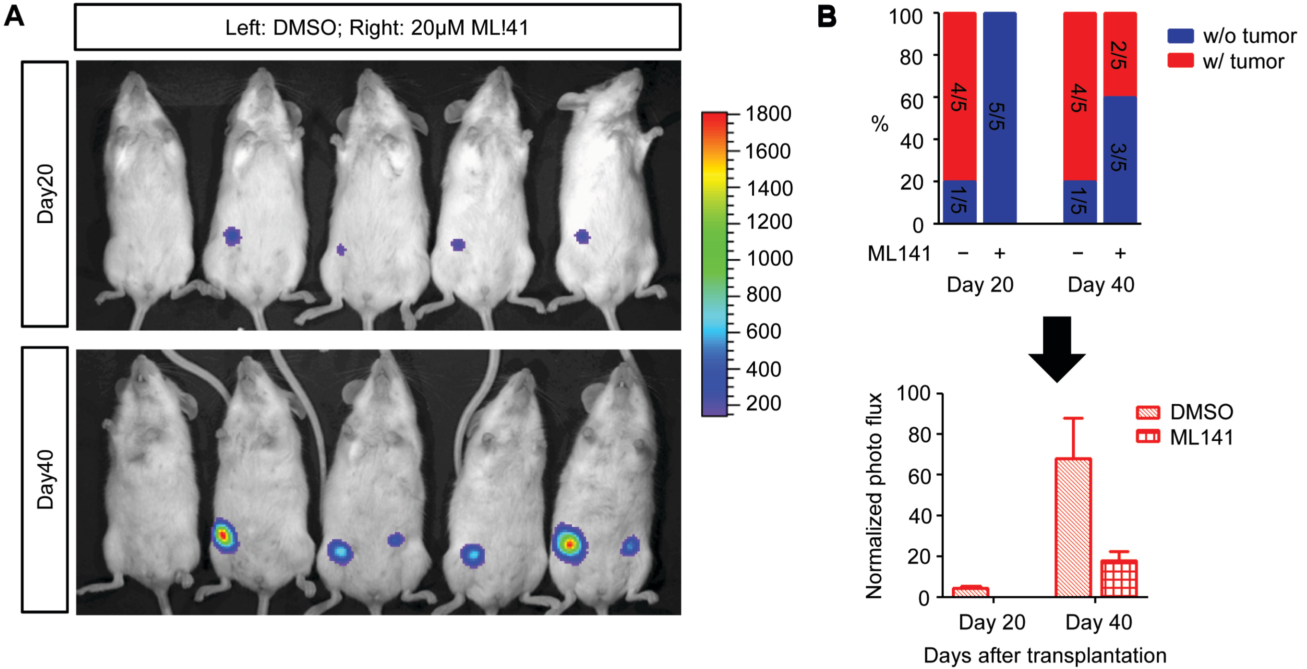

Supporting information Fig 11. ML141 pretreatment decreases frequency of tumor formation of BLBCs. For each xenograft, 10,000 cells were transplanted into mammary fat pads of a female NOD/SCID mouse. (A) 40 hour DMSO or 20µM ML141-pretreated luciferase expressing MDA-MB 231 cells were transplanted on left and right sides of animals, respectively. Tumor sizes were tracked and recorded by live imaging every 20 and 40 days after transplantation. (B) The number on either side of animals with tumor formation was recorded, and luciferase intensity of tumors was measured and qualified in the graph.

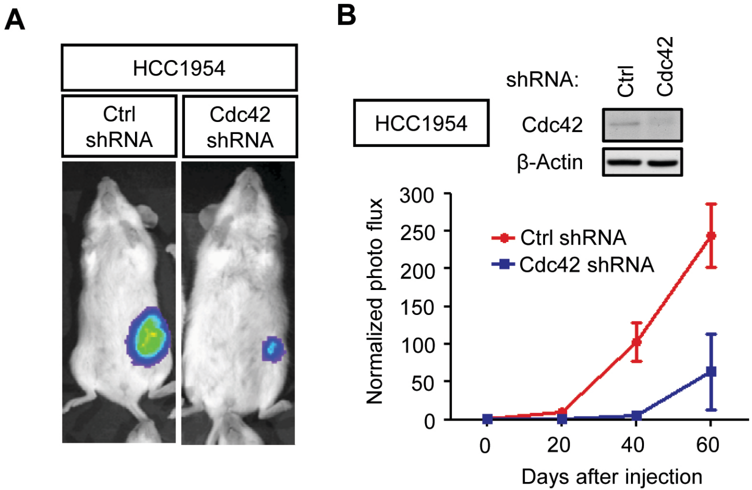

Supporting information Fig 12. Cdc42 knockdown suppresses tumor growth of BLBCs *in vivo*. For each xenograft, 100,000 cells were transplanted into mammary fat pad of a female NOD/SCID mouse. (A) Scrambled or Cdc42 knockdown HCC1954 cells were transplanted, and tumor sizes were tracked and recorded by live imaging over time till 60days after transplantation. (B) Luciferase intensity was measured and qualified in the graph.

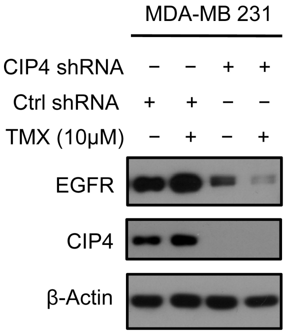

Supporting information Fig 13. CIP4 knockdown enables TMX to cause reductions in EGFR levels. MDA-MB 231 cells expressing scrambled or CIP4 shRNAs were exposed to DMSO or 10μM TMX for 4 hours, after which cells were harvested for measurement of levels of EGFR, CIP4 and β-Actin.

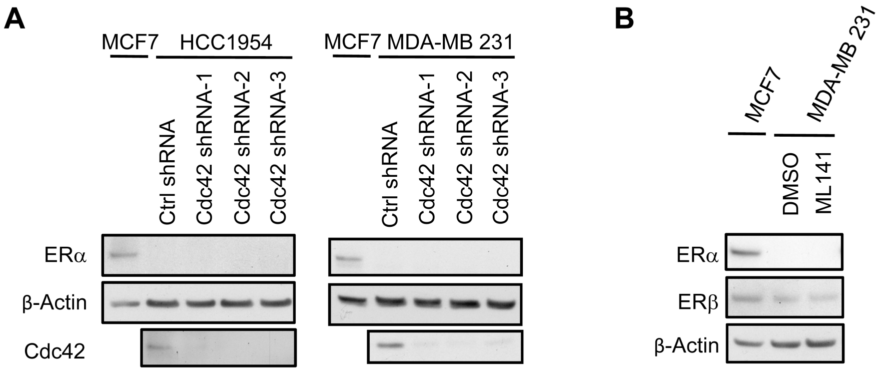

Supporting information Fig 14. Expression of ERs is not altered upon knockdown or inhibition of Cdc42. (A) ERα expression was measured by immunoblotting of HCC1954 and MDA-MB 231 cells expressing shRNAs for Cdc42. (B) MDA-MB 231 cells treated with DMSO or with 20μM ML141 (the concentration enabling TMX killing; Fig 3) for 48 hours, after which cells were lyzed and then examined for expression of ERα and ERβ by Western Blot.
